# Molecular mechanisms of low-temperature sensitivity in tropical/subtropical plants: a case study of *Casuarina equisetifolia*

**DOI:** 10.48130/FR-2023-0020

**Published:** 2023-08-31

**Authors:** Huimin Ren, Yue Zhong, Liangyu Guo, Jamshaid Hussian, Chen Zhou, Youzhi Cao, Wenwu Wu, Shenkui Liu, Guoning Qi

**Affiliations:** 1 State Key Laboratory of Subtropical Silviculture, School of Forestry and Biotechnology, Zhejiang A & F University, Hangzhou 311300, Zhejiang, China; 2 Department of Biotechnology, COMSATS University Islamabad, Abbottabad Campus, 22060, University Road Abbottabad, Pakistan; 3 Institute of Functional Nano & Soft Materials (FUNSOM), Soochow University, Suzhou 215123, Jiangsu, China

**Keywords:** Cold stress, Cold acclimation, Transcriptome, CBF, Cold responsive gene, *Casuarina equisetifolia*

## Abstract

Low temperature is a limiting factor affecting plant growth and development. *Casuarina equisetifolia*, a typical tropical and subtropical tree important for the ecological restoration of coastal beaches, is sensitive to cold stress. By comparing cold tolerance between *C. equisetifolia* and *Arabidopsis*, we investigated the molecular basis underlying the cold sensitivity of *C. equisetifolia*. Transcriptomic analysis showed that the number of cold-induced genes in *C. equisetifolia* was significantly less than that in *Arabidopsis*, and notably, the response of cold-induced genes was also delayed in *C. equisetifolia*. Among the cold-induced genes, *C-repeat binding factors* (*CBFs*), the major transcription factors in cold acclimation in *Arabidopsis*, showed a delayed cold-induced expression in *C. equisetifolia*, despite that *C. equisetifolia CBFs* could restore the low temperature-sensitive phenotype of *Arabidopsis* *cbfs* triple mutants. Interestingly, some key cold-responsive genes (e.g., *COR15A* and *COR15B*) targeted by *Arabidopsis CBF* were absent in the *C. equisetifolia* genome and many cold-responsive genes in *C. equisetifolia* lacked the DRE element (i.e., CBF binding *cis*-element). Moreover, like in *C. equisetifolia*, many *COR* genes in other tropical/subtropical plants lacked the DRE element or were directly missing. These two factors could be the underlying reasons for the low-temperature sensitivity of *C. equisetifolia* and other tropical/subtropical plants.

## Introduction

Low temperature is one of the main factors restricting plant growth, development and geographic distribution. Cold stress is categorized as chilling (0−15 °C), generally suffered by tropical and subtropical plants, and freezing (< 0 °C), experienced by temperate plants^[1]^. The adaptability of plants to chilling and freezing is termed as chilling resistance and freezing resistance, respectively.

To adapt to cold climates, plants have developed a set of complex mechanisms^[2]^. Lots of temperate plants increase their freezing tolerance through cold acclimation (CA). The molecular mechanism of cold acclimation in *Arabidopsis* has been well studied, and a relatively clear cold regulatory network has been established. The CBF/DREB1 (C-REPEAT BINDING FACTOR/DEHYDRATION-RESPONSIVE ELEMENT-BINDING PROTEIN 1)-dependent transcriptional modulation pathway takes indispensable parts in plant response to both chilling and freezing^[3]^. In the CBF-dependent pathway, the expression of *CBF* genes are rapidly and significantly up-regulated by low temperature, and the corresponding CBF proteins activate the cold-regulated (*COR*) genes' expression through specific binding to the C-repeat/DRE *cis*-elements (G/ACCGAC) motif contained in the promoters of *COR* genes, resulting in the improvement of cold acclimation and freezing tolerance^[4−6]^. Moreover, the deletion of the C-repeat/DRE regulatory motif of *AtCBF2* decreases the cold tolerance of plants^[7]^.

CBFs function as a molecular switch under low temperature stress and is highly conserved in different higher plants. The transgenic plants with the overexpressing of endogenous *CBFs* represent distinctly improved tolerance to freezing^[8−12]^. In *Arabidopsis*, CBF1 (DREB1B), CBF2 (DREB1C), and CBF3 (DREB1A) are highly similar in sequence and redundant in function. The triple mutants of *CBF1* *CBF2* *CBF3* (*cbf1 cbf2 cbf3*) fail to respond to chilling stress and are hypersensitive to freezing stress after cold acclimation^[13]^. Previous research reported that the expression of *CBFs* is directly modulated by some other transcription factors, such as Inducer of *CBF* expression 1 (ICE1)^[14−16]^, Calmodulin-binding Transcription Activators (CAMTAs)^[17,18]^, MYB15^[19]^, Ethylene-Insensitive 3 (EIN3), Brassinazole-Resistant 1/BRI1-EMS-SUPPRESSOR1 (BZR1/BES1)^[20−22]^, Phytochrome-Interacting Factors 3/4/7 (PIF3/4/7)^[23,24]^, and Circadian Clock-Associated 1/Late Hypocotyl (CCA1/LHY)^[25,26]^. In addition, some genes independent CBF pathway have also been identified to be involved in cold response, such as *HY5* (*ELONGATED HYPOCOTYL 5*)^[27]^ and *HSFA1*^[28]^.

Plants from temperate-zones exhibit a strong resistence to low temperature conditions, whereas plants originating from the tropics and subtropics are generally sensitive to the chilling temperatures, and undergo irreversible damage^[29−31]^. Many studies have investigated the physiological and biochemical response of tropical and subtropical plants under cold stress, but, besides identifying some cold responsive genes, limited systematic research has been conducted to understand the reasons for the tropical and subtropical plants sensitivity to cold conditions. For example, it has been shown that the CBF-dependent pathway could perform its functions in rubber tree (*Hevea brasiliensis*)^[32]^ and some genes involved in cold adaptability, such as *HbICE1*^[33]^, *HbCOR47*^[34]^ and *HbEBP*1^[35]^ have been characterized. Compared to Cavendish (a cultivar of banana), the stonger cold resistence of another specie Dajiao could be due to the quick activation and induction of *ICE1* and *MYBS3*^[36]^. In Moso bamboo, *PeDREB1* is highly up-regulated by cold and *PheMYB4-1* also mediated the response of bamboo to cold^[37,38]^. Further transcriptome analysis under different cold conditions in Moso bamboo revealed that most of the genes involved in cold response are late-responsive ones^[39]^. So, it is very important to improve the cold resistence of tropical and subtropical economic tree species through elucidating the underlying basis of intolerance to cold stress at the molecular level.

*Casuarina equisetifolia* (*C. equisetifolia*) is a drought-, salinity-, and barren-tolerant tree species, and native to southeastern Australia^[40]^. It is a typical tropical plant with poor cold tolerance with 22.1−26.9 °C being the suitable temperature for its growth^[41]^. In this study, *C. equisetifolia* was used as a typical example of tropical and subtropical tree. We compared transcriptome of *C. equisetifolia* to that of *A. thaliana*, in which a clear view of the cold signal network has been investigated, under cold stress for different treatment times to identify the factors causing the cold intolerance in *C. equisetifolia.*

## Materials and methods

### Plant materials and low temperature treatments

The seeds of *C. equisetifolia* were sown in the pot and grown in greenhouses for two months and then the chilling treatment was carried out. *A. thaliana* seeds of wild-type (Col), mutants and transgenic plants were placed on Murashige and Skoog (MS) medium for 7 d at 25 °C with 16 h/8 h light/dark cycle and then transferred into the greenhouse. After two weeks, the seedlings of *A. thaliana* were exposed to low temperatures.

The seedlings of both *C. equisetifolia* and *A. thaliana* were firstly treated at 4 °C for 7 d for survival rate analysis and at 4 °C for 4 d for ion leakage experiment in a climate-controlled chamber, respectively, and then transferred to −8 °C for 6 h. Subsequently, the treated seedlings were recovered at 4 °C for 12 h and then moved to greenhouses for 7 d. Finally, the survival and ion leakage rates were calculated.

### Ion leakage rate determination

Leaves (100−200 mg) were detached from cold-treated and untreated *A. thaliana* and *C. equisetifolia*, respectively, and were washed three times using deionized water. The samples were then placed on the clean glass chambers with 10 mL deionized water and were shaken at room temperature for 1 h to mix. The electrical conductivity (C1) of the solution was detected using a conductivity meter (DDS-307W, Gallop). Subsequently, the chambers were boiled in a water bath for 20 min, followed cooling for 1 h. After that, the electrical conductivity (C2) of the solution in each tested chamber was surveyed. The relative electrolyte leakage (REL) was obtained based on the formula REL = C1/C2 × 100%. The experiments were performed three times and the data were represented as the means ± standard deviation (SD) of three independently biological replicates.

### Analysis of cold-induced transcriptomic changes in *A. thaliana* and *C. equisetifolia*

In order to identify the differential transcriptomic changes between *A. thaliana* and *C. equisetifolia* under cold stress, 21-day old *A. thaliana* and two-month old *C. equisetifolia* seedlings were used for low temperature treatments, which were 4 °C for 0, 2, and 24 h for *A. thaliana* and 0, 10 min, 2 h, 24 h, and 168 h for *C. equisetifolia*. Total RNA was isolated from the treated seedlings and evaluated by NanoDrop ND-1000. Poly (A)-RNA was enriched for constructing cDNA library and sequencing by Illumina NovaSeq 6000 (2 × 150 bp paired-end reads). Each time point contained three replicates.

In order to analyze the RNA-seq dataset, we designed a pipeline. In brief, we aligned the clean reads of the samples to the corresponding genome and reference genes using hisat2-2.1.0^[42]^ with the parameters (--min-intronlen 20 --max-intronlen 5,000 --rna-strandness RF). Gene expression in the normal and after the onset of cold treatment was determined using StringTie v2.0.3^[43]^. Trimmed Means of M values (TMM) values were used to represent gene expression value. The cold-induced genes were defined with more than two-fold expression under cold stress relative to that under normal conditions.

### GO enrichment analysis of cold-induced genes

The GO term annotations of *A. thaliana* genes were directly downloaded from the website The *Arabidopsis* Information Resource (www.arabidopsis.org) (TAIR10)^[44]^. As reported in previous research^[45,46]^, the protein coding genes of *C. equisetifolia* were annotated with the highest similarity to *A. thaliana* proteins through BLASTP searches (E-value < 1e–5). The GO enrichment analysis of cold-induced genes was performed through the OmicShare tools (www.omicshare.com/tools).

### *Cis*-regulatory binding motifs analysis of *CBFs*

To explore the difference of CBF-CORs-mediated cold signaling pathway between *A. thaliana* and *C. equisetifolia*, the following pipeline was developed. First, we collected the well-known CBF regulons with cold-elevated expression in *A. thaliana* from previous report^[47]^. Next, we identified the orthologous of those CBF regulons in *C. equisetifolia* using BLASTP program (E-value < 1e–10). Then, we extracted 1- and 2-Kb upstream promoter sequences of the regulons in *A. thaliana* and *C. equisetifolia* from the corresponding genomes, respectively. Besides, the published *A. thaliana* CBFs *cis*-regulatory motifs (G/ACCGAC) from early research were organized^[47,48]^. Using the modules as query, those promoters were searched by FIMO^[49]^. Finally, the CBF binding sites in 1- and 2-Kb upstream promoter sequences of CBF regulons were identified.

### qPCR validation of cold-related genes

This study utilized quantitative reverse transcription PCR (qPCR) following the methodology described by Cheng et al.^[35]^. The primers of genes for quantification were designed using Primer 5 software and listed in Supplemental Table S1. The primers were synthesized by Hangzhou Youkang Biotechnology Co., Ltd (Hangzhou, China). qPCR was performed using the ChamQTM SYBR Color qPCR Master on a Bio-Rad Iq5 RT-PCR instrument, following the experimental protocols outlined in the instruction manual. The data was analyzed using 2^−^^ΔΔCᴛ^ method.

### Transgenic plant generation

The full-length coding sequences (CDS) of *CeqCBF* genes (*CeqCBF1* and *CeqCBF3*) were amplified and cloned into the pCAMBIA1300-35S::GFP vector between the Sma I and Xba I sites, termed as *35S::CeqCBF1OE/cbfs* and *35S::CeqCBF3OE/cbfs.* The recombinant plasmid was transformed into *Agrobacterium tumefaciens EHA105*. The transgenic lines were generated through *Agrobacterium*-mediated floral-dipping method and screened by Hygromycin B. The seeds from T_3_ homozygous transgenic plants were used for phenotype detection under low temperature conditions. The primers used are listed in Supplemental Table S2.

## Results

### Differences in cold resistance of *A. thaliana* and *C. equisetifolia*

**Figure 1:**
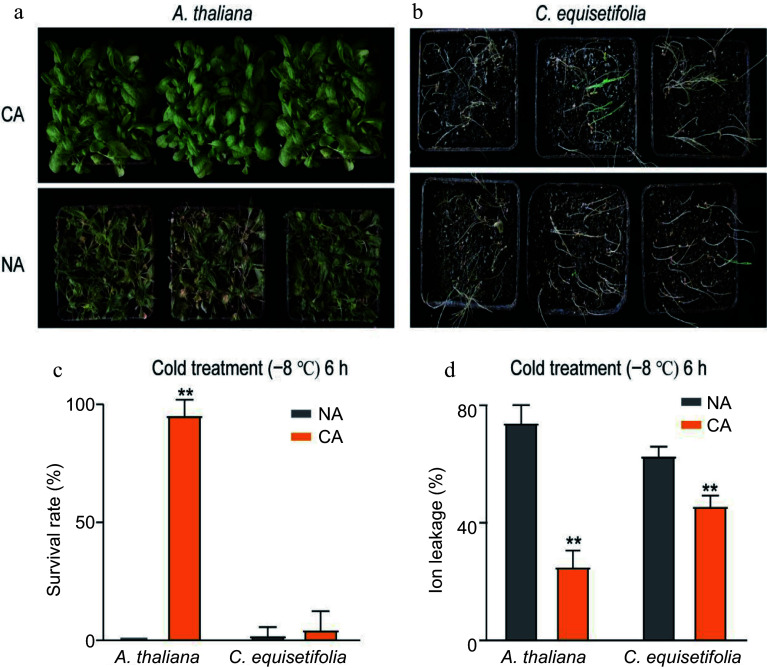
Analysis of cold resistance of *A. thaliana* and *C. equisetifolia.* (a), (b) Freezing phenotypes (c) survival rates, and (d) ion leakage, of three-week-old wild type *A. thaliana* and two-month-old *C. equisetifolia*. The seedlings were grown under a 16-h-white light/8-h-dark photoperiod (LD) at 25 °C and either directly exposed to freezing treatment (non-acclimated, NA), or treated at 4 °C for 4 d or 7 d before freezing treatment (cold-acclimated, CA). Representative photographs were taken after a 3-d recovery period, survival rates were calculated, and the ion leakage was measured. Data are presented as the mean ± standard deviation of mean of three biological replicates. Asterisks represent significant differences compared to the non-acclimated plant. ** *p* < 0.01.

The molecular regulation mechanism of *A. thaliana* in response to cold stress is well documented*.* To investigate the cold stress response of *C. equisetifolia*, we compared the differences in cold response between *A. thaliana* and *C. equisetifolia*, and determined the impact of cold acclimation on improving cold resistance in these plant species. The morphological performance of both *A. thaliana* and *C. equisetifolia* with (4 °C for 7 d and then treated at −8 °C for 6 h) and without (directly exposed to −8 °C) cold acclimation was significantly different. Compared to the cold-acclimated (CA) counterparts, plants (both *A. thaliana* and *C. equisetifolia*) non-acclimated (NA) showed typical cold injury symptoms like desiccated foliage and eventually almost all plants died (Fig. 1a, b). Moreover, *C. equisetifolia* displayed higher cold sensitivity than *A. thaliana* (Fig. 1b). The survival rate of CA plants (both *A. thaliana* and *C. equisetifolia*) was higher than that of NA plants. In detail, the survival rate of *A. thaliana* achievd 95.8% after cold acclimation for 7 d, whereas the survival rate of *C. equisetifolia* was about 4.93% (Fig. 1c), indicating that cold acclimation could improve the cold resistence of plants and *C. equisetifolia* had a weaker cold acclimation capacity than *A. thaliana*.

The relative electrolyte leakage is a vital indicator of membrane permeability. So, the electrolyte leakage assay was carried out in both plants with and without cold acclimation based on the method reported by Jiang et al.^[50]^. The data showed that the electrolyte leakage of CA plants was dramatically lower than that of the NA counterparts (Fig. 1d). Moreover, the influence of cold acclimation on electrolyte leakage was more obviously in *A. thaliana* (dropped from 74.2% to 24.5%) than in *C. equisetifolia* (dropped from 62.9% to 45.8%) (Fig. 1d).

### Differential expression patterns induced by chilling stress

To further investigate the transcriptional response of *C. equisetifolia* under cold stress, *C. equisetifolia* and *A. thaliana* wild-type (Col) seedlings were exposed to a cold treatment at 4 °C for different time periods (0, 10 min, 2 h, 24 h, 168 h for *C. equisetifolia*, and 0, 2 h, 24 h for *A. thaliana*). Leaves from the treated seedlings were harvested at indicated time points and 24 cDNA libraries (15 for *C. equisetifolia* and nine for *A. thaliana*) were prepared which were subjected to paired-end sequencing. According to Pearson Correlation Coefficient (PCC), the sample replicates were clustered closely and independently, illustrating that the results were highly reproducible with good quality (Supplemental Fig. S1).

To identify the genes up-regulated by cold stress, the expression of a particular gene was considered as cold-induced with log2 (fold-change) ≥ 1 (*p* value < 0.01) in comparison with control. The genes with read counts < 5 in each sample were removed.

**Figure 2:**
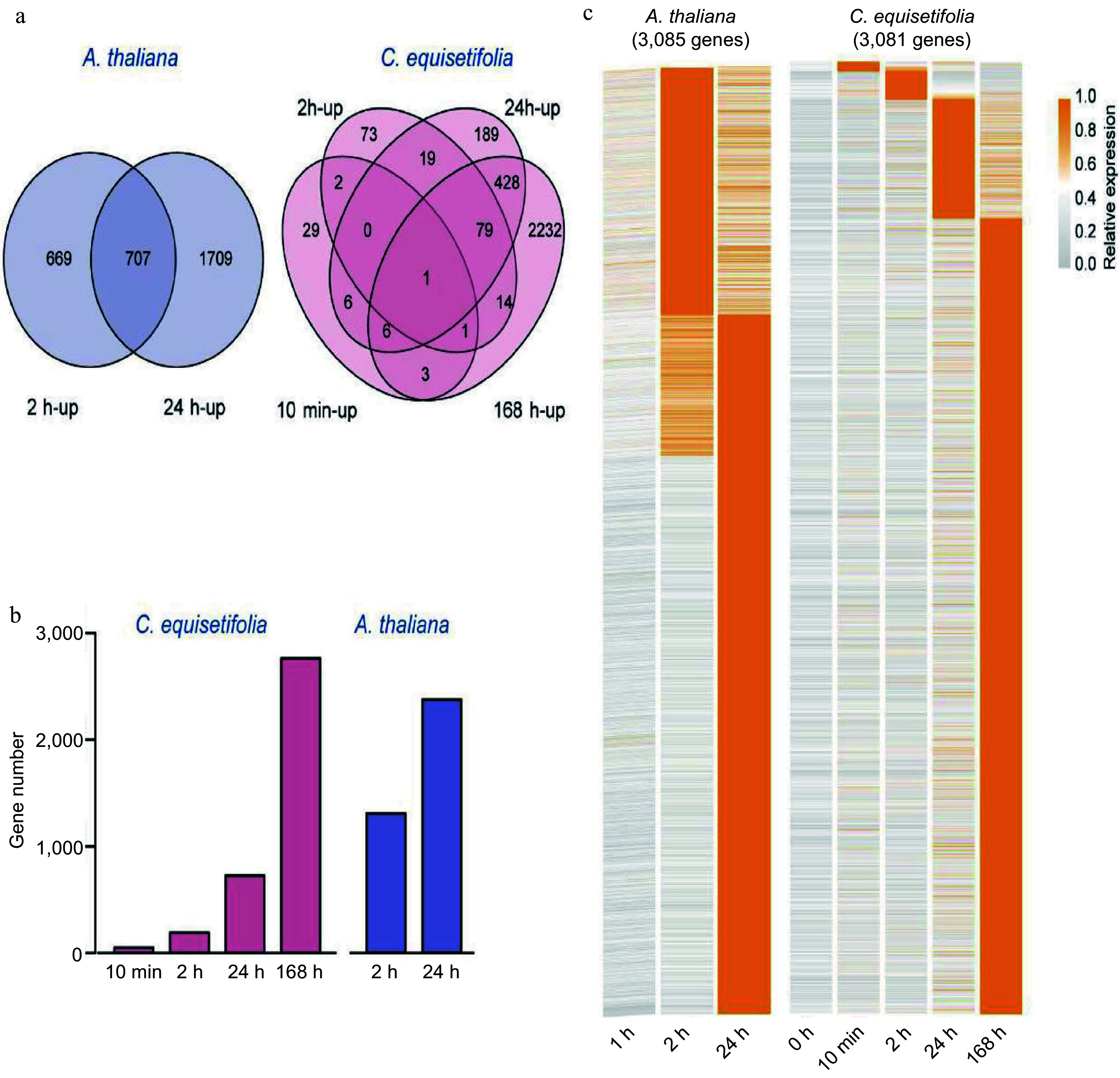
Comparison of the number of cold-induced genes at different time points in *A. thaliana* and *C. equisetifolia.* (a) Venn diagram illustrating the cold-induced genes at 10 min, 2, 24 and 168 h (*C. equisetifolia*) and 2 h, 24 h (*A. thaliana*) after chilling treatment. (b) The number of cold-induced genes between the different plants and time points. (c) Expression profiles and clustering of 3,081 cold-induced genes in *C. equisetifolia* and 3,085 cold-induced genes in *A. thaliana* at different times after cold acclimation.

According to the Venn diagram, a total of 3,085 (1,376 at 2 h, 2,416 at 24 h) cold-induced genes were identified in *A. thaliana*, whereas a total of 3081 genes were up-regulated by chilling in *C. equisetifolia* (48 at 10 min, 189 at 2 h, 728 at 24 h, and 2,764 at 168 h) (Fig. 2a). Further, we found that there was a considerable difference in the number of cold-induced genes at each treatment time point between *C. equisetifolia* and *A. thaliana*, such as 189 vs 1,376 at 2 h, 728 vs 2,416 at 24 h, while the number of cold-induced genes at 168 h in *C. equisetifolia* was close to the number at 24 h in *A. thaliana* (Fig. 2b), displaying an obvious delay in cold response in *C. equisetifolia*. Moreover, expression profiles and clustering of cold-induced genes showed that numerous genes in *A. thaliana* was significantly induced by cold at 2 and 24 h after the start of cold treatment, while only fewer genes were up-regulated in *C. equisetifolia* at these time points, indicating that *C. equisetifolia* may require a more prolonged cold treatment than *A. thaliana* for transcriptome response (Fig. 2c).

### Cold-induced genes GO enrichment assay

**Figure 3:**
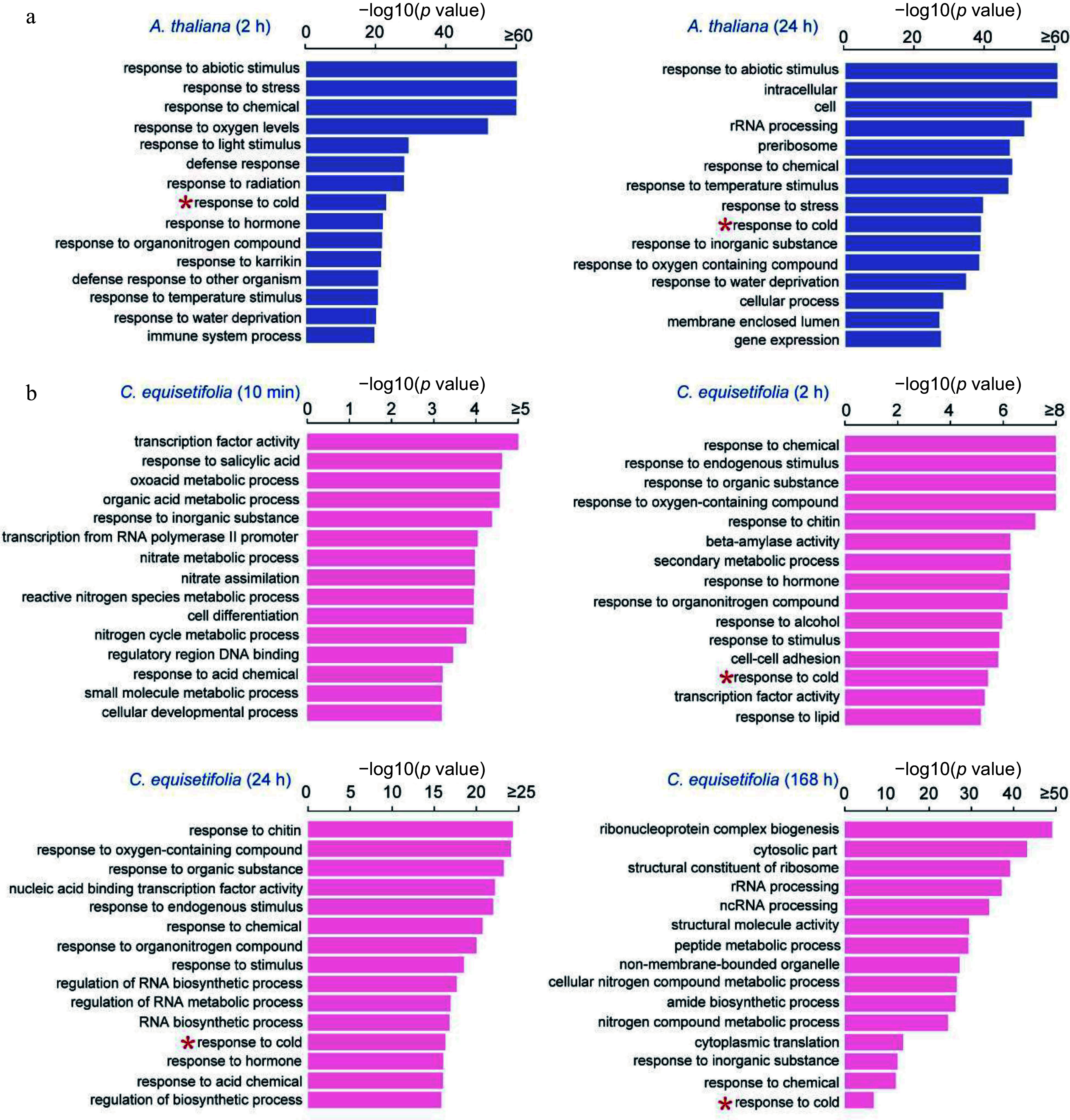
GO enrichment analysis of cold-induced genes at different time points in *A. thaliana* and *C. equisetifolia* under 4 °C cold treatment. (a) *A. thaliana,* (b) *C. equisetifolia.*

GO enrichment analysis was carried out to classify the putative functions of cold induced genes. The top 15 highly enriched functional groups are shown in Fig. 3. For *A. thaliana*, 15 functional groups and 11 functional groups separately exhibited significant enrichment within the biological processes category during the 2 h and 24 h of cold stress. These enriched groups included response to abiotic stimulus, cold, temperature stimulus, water deprivation and so on (Fig. 3a).

For *C. equisetifolia*, 13 functional groups were significantly enriched at the 10 min time point within the biological processes category, and two functional groups were enriched within the molecular process. For the 2 h time point, enriched biological processes included response to endogenous stimulus, chemical, and cold, etc., while the enriched molecular processes included beta-amylase activity and transcription factor activity. At the 24 h time point, 14 groups were enriched within the biological processes category. And at 168 h after the initiation of chilling, the GO enrichment analysis revealed that 11 functional groups were enriched in the category of biological processes (Fig. 3b). It is worth mentioning that, with the exception of the 10 min time point in *C. equisetifolia,* biological process 'response to cold' was represented in both *C. equisetifolia* and *A. thaliana* at all cold treatment time points, indicating the induction of cold response at the transcriptional level.

To gain further insights, the differences in GO entries between *A. thaliana* and *C. equisetifolia* at 2 h and at 24 h treatment time points were analyzed, respectively. The results showed that GO entries related to stress response, such as hormone, abiotic stimulus, cold, and water deprivation were dramatically enriched in *A. thaliana* at 2 h, whereas in *C. equisetifolia* at 24 h (Supplemental Fig. S2a). Furthermore, at each time point, the top 200 enriched GO entries were examined and found that the two species shared 115 GO entries at 2 h of *A. thaliana* and 24 h of *C. equisetifolia*, as well as at 24 h of *A. thaliana* and 168 h of *C. equisetifolia* (Supplemental Fig. S2b). These results implied that though the cold stress response of *C. equisetifolia* was comparatively delayed, the overall biological enrichment classes are similar to *A. thaliana*.

### Comparison of *C. equisetifolia* and *A. thaliana* GO enriched cold-response entry genes

**Figure 4:**
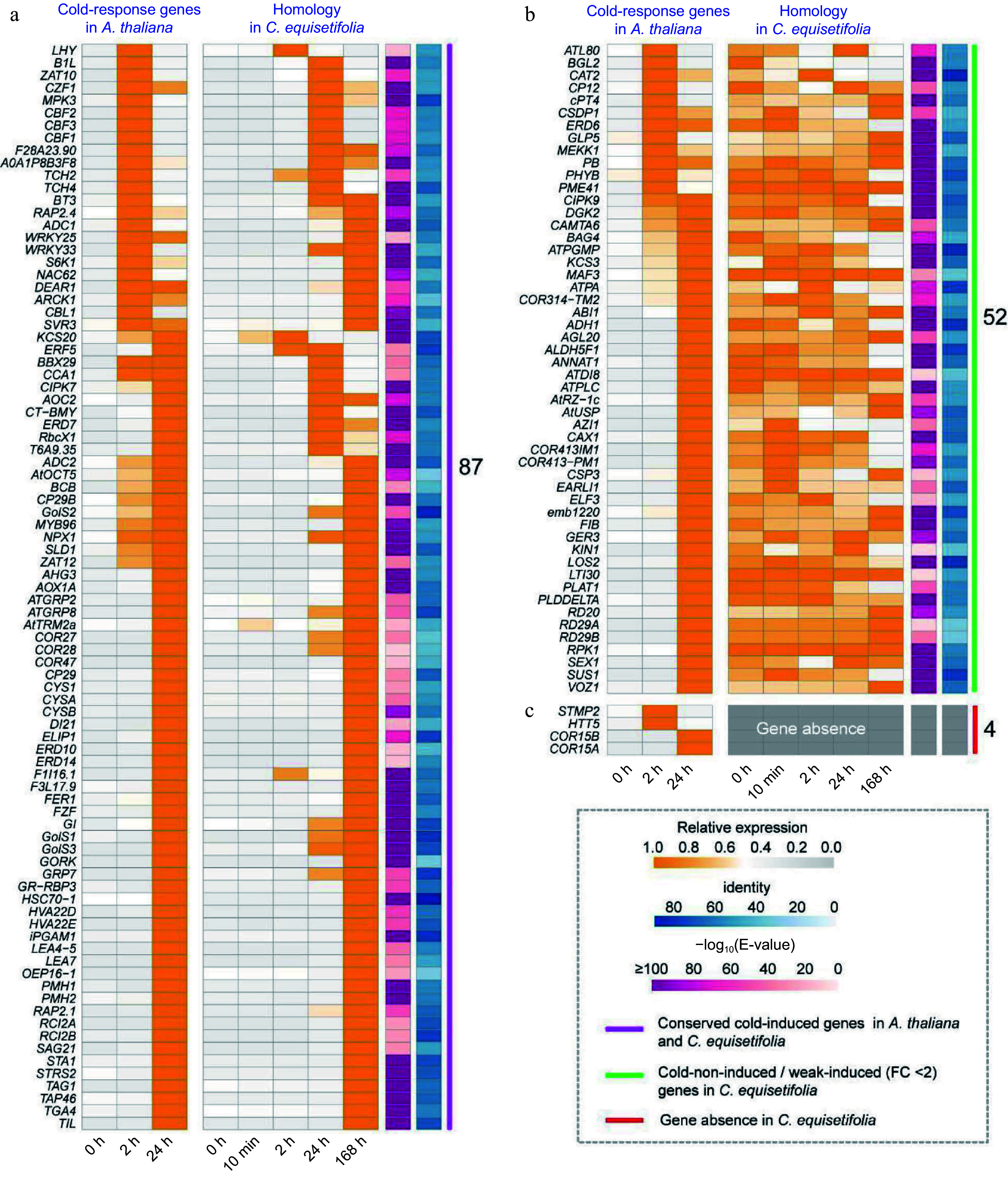
Comparison of the 'cold response entries' in *A. thaliana* and *C. equisetifolia*. (a) Conserved cold-induced genes in *A. thaliana* and *C. equisetifolia.* (b) Unresponsive to cold stress/ weakly cold-induced (FC < 2) genes in *C. equisetifolia*. (c) Gene absent in *C. equisetifolia.*

As plant cold stress tolerance is a polygenic controlled trait^[51]^, an array of genes and proteins play vital roles in cold tolerance^[52−55]^. To analyze the cold response pathway in *C. equisetifolia*, we extracted cold induced genes from *A. thaliana* transcriptome and then identified the homologous genes in *C. equisetifolia* using *A. thaliana* cold-induced genes as the query. A total of 139 and 143 genes were identified in *C. equisetifolia* and *A. thaliana*, respectively, in which 87 were significantly up-regulated by low temperature both in *A. thaliana* and in *C. equisetifolia* (Fig. 4a). However, the expression of most of them in *C. equisetifolia* was dramatically increased at 168 h and obviously later than that in *A. thaliana*, which were induced at 24 h (Fig. 4a), inferring that their delayed expression may influence the cold tolerance of *C. equisetifolia*. In addition, 52 genes had no change in transcription level under cold conditions in *C. equisetifolia* but up-regulated *A. thaliana* (Fig. 4b). Some of these genes have been reported to play notable roles in response to cold, such as *RD29A/B*^[56]^*, COR413PM1*^[57,58]^*, KIN1*/*KIN2* (ABA-inducible protein-coding), *SUS1, DAG2,* and *LOS2*^[47]^. Besides, four genes namely *STMP2* (Salt Tolerance-Associated Membrane Protein 2), *HTT5* (HEAT-INDUCED TAS1 TARGET 5), *COR15A* (Cold regulated 15A), and *COR15B* were absent in the transcriptome of *C. equisetifolia* (Fig. 4c)*.*

Based on the above analysis, we postulated three reasons for relative cold sensitivity of *C. equisetifolia*. First, compared with *A. thaliana*, the expression of cold responsive genes was delayed. Second, partial key cold-response genes were not up-regulated under cold stress, and last but not least, several functional cold responsive genes reported in *A. thaliana* were absent in *C. equisetifolia*.

### Confirmation of the expression profiles of selected cold response genes by qPCR in *C. equisetifolia*

**Figure 5:**
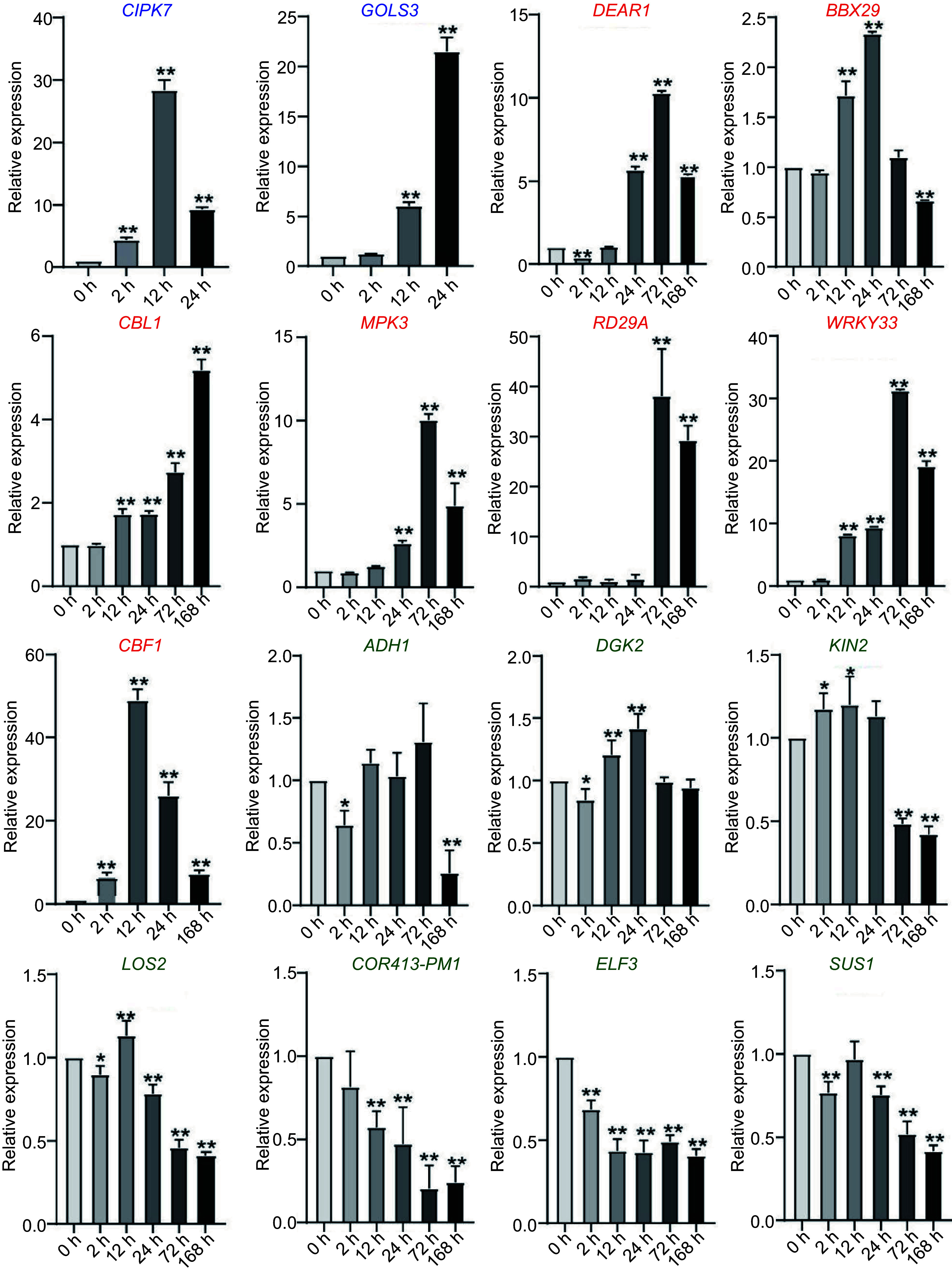
Expression of cold-response genes in *C. equisetifolia*, containing two similar expression pattern genes (*CIPK7*, *GOLS3*), seven delayed genes (*DEAR1*, *BBX29*, *CBL*, *MPK3*, *RD29A*, *WRKY33*, *CBF1*), four weakly responsive genes (*ADH1*, *DGK2*, *KIN1*, *LOS2*) and three non-responsive genes (*COR413-PM1*, *ELF3*, *SUS1*). *CIPK7*, CBL-interacting protein kinase 7; *GOLS3*, galactinol synthase 3; *DEAR1*, cooperatively regulated by ethylene and jasmonate 1; *BBX29*, B-box type zinc finger family protein; *CBL*, calcineurin B-like protein 1; *MPK3*, mitogen-activated protein kinase 3; *RD29A*, low-temperature-responsive protein 78 (LTI78); *WRKY33*, *WRKY* DNA-binding protein 33; *ADH1*, alcohol dehydrogenase 1; *DGK2*, diacylglycerol kinase 2*; KIN1*, stress-responsive protein (KIN1)/stress-induced protein (KIN1); *LOS2*, involved in light-dependent cold tolerance and encodes an enolase; *COR413-PM1*, cold regulated 413 plasma membrane1; *ELF3*, hydroxyproline-rich glycoprotein family protein; *SUS1*, encodes a protein with sucrose synthase activity. The values are the mean ± standard deviation of three biological replicates. Relative expression in untreated plants (0 h) was set to 1. *p < 0.05, **p < 0.01.

To confirm the expression profiles of cold responsive genes in *C. equisetifolia*, the transcriptional level of some genes was analyzed through qPCR. We selected two genes with the similar expression pattern to *A. thaliana* (*CIPK7*, *GOLS3*), seven genes with delayed cold-induced expression (*DEAR1*, *BBX29*, *CBL1*, *MPK3*, *RD29A*, *WRKY33, CBF1*), and seven genes with no change in expression (*ADH1*, *DGK2*, *KIN1, LOS2*, *COR413-PM1*, *ELF3*, *SUS1*) in *C. equisetifolia* under cold stress. The qPCR results revealed that the expression of *CIPK7* and *GOLS3* was markedly induced under cold conditions at 2 h and 24 h in *C. equisetifolia*, which aligned with the transcriptome data of *A. thaliana* (Fig. 5). On the other hand, the expression of *DEAR1*, *BBX29*, *CBL1*, *MPK3*, *RD29A*, *WRKY33,* and *CBF1* in *C. equisetifolia* was prominently up-regulated at 168 h, whereas in *A. thaliana*, the up-regulation was observed a significant delay at 24 h (Fig. 5). Furthermore, the expression of *ADH1*, *DGK2*, *KIN1*, *LOS2*, *COR413-PM1*, *ELF3*, and *SUS1* was not increased by cold stress in both species, and some of these genes were even down-regulated (Fig. 5). In addition, the expression of *ADH1*, *DGK2*, *KIN1*, *LOS2*, *COR413-PM1*, *ELF3*, and *SUS1* was not induced by cold stress, instead a few of these were down-regulated (Fig. 5). These findings demonstrated that the qPCR results for the selected genes in *C. equisetifolia* were consistent with the transcriptome data of both *A. thaliana* and *C. equisetifolia*.

### Functional characterization of *CBFs* in *C. equisetifolia*

**Figure 6:**
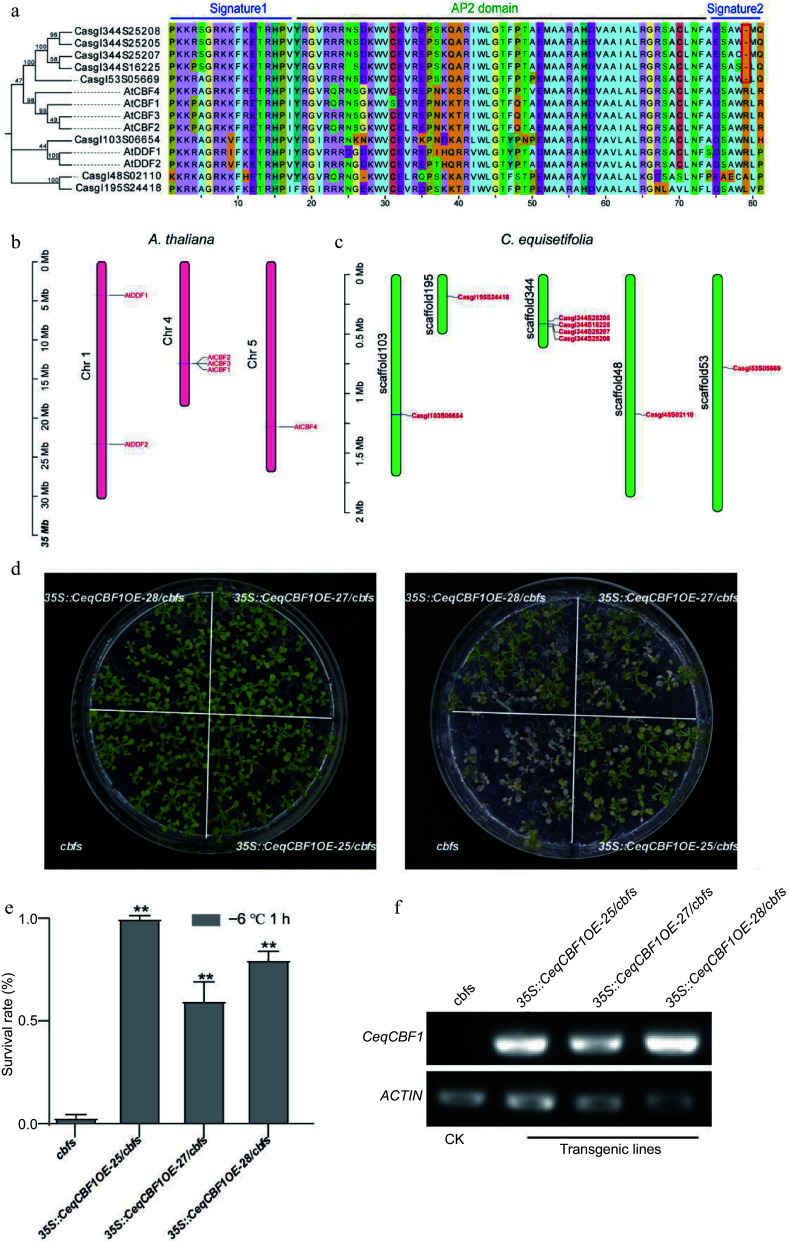
CBF in *C. equisetifolia* was involved in cold regulation. (a) Alignment of AP2/ERE domain amino acid sequences between *A. thaliana* and *C. equisetifolia.* (b), (c) Chromosome positions of *CBF* genes. (d) Freezing phenotypes, (e) survival rates and (f) the expression of *CeqCBF1* in *A. thaliana* *cbf* triple mutants and transgenic lines with overexpressing of *CeqCBF1*. Two-week-old plants grown on MS plates at 22 °C were treated at −6 °C for 1 h after cold acclimation at 4 °C for 3 d. Asterisks indicate significant differences compared to the cbfs mutant plants. **p < 0.01.

It is well known that the C-repeat binding factor (CBF) plays a predominant role in cold acclimation to gain the maximum cold resistence in plants^[5,59]^. We identified the Arabidopsis homologous of CBFs in *C. equisetifolia* and descovered that the transcriptional level of *CeqCBF1* was up-regulated by low temperature (Fig. 5). To verify whether cold intolerance of *C. equisetifolia* was associated with CBFs, we identified CBF family in *C. equisetifolia.* There were eight CBF homologous proteins in the transcriptome of *C. equisetifolia* by BLASP, and we constructed the phylogenetic tree to determine the evolutionary relationship of *C. equisetifolia* CBFs (Fig. 6a). Further, the conserved structure domains of CBFs were aligned. The results showed that signature1 and AP2 domain were highly conserved, but an amino acid R was absent from signature2 in *C. equisetifolia* and the first amino acid D in signature2 in *A. thaliana* was changed into E (ESAW-) in *C. equisetifolia* (Fig. 6a), inferring that it may impact CBFs' function in cold response pathway. The chromosome localization data showed that *A. thaliana* *CBF1*, *CBF2*, and *CBF3* are arranged in series on chromosome 4 (Fig. 6b), which was consistent with the previous research^[60,61]^. The analysis of *CeqCBF* genes localization on the chromosomes in *C. equisetifolia* revealed that four out of eight genes were found in tandem arrangements (Fig. 6c).

To study the function of *CeqCBFs* in response to cold stress, the recombination plasmids were separately constructed with *CeqCBF1* (Casgl53S05669) (exhibiting high homology to AtCBF1/AtCBF2/AtCBF3) and *CeqCBF3* (Casgl344S25208) under 35S promoter and then transferred into *Arabidopsis cbfs* triple mutants (*cbf1cbf2cbf3*) by floral dipping method. The results of phenotype analysis showed that the transgenic lines with *CeqCBF1* overexpression (*35s::CeqCBF1OE*-25/*cbfs*, *35s::CeqCBF1OE*-27/*cbfs*, *35s::CeqCBF1OE*-28/*cbfs*) and *CeqCBF3* overexpression (*35s::CeqCBF3OE*-20/*cbfs*, *35s::CeqCBF3OE*-27/*cbfs*, *35s::CeqCBF3OE*-40/*cbfs*) in the *cbfs* triple mutants background could restore the sensitivity of *cbfs* triple mutants to cold stress to a certain extent and display the increased cold tolerance, respectively (Fig. 6d, Supplemental Fig. S3a). The survival rate of transgenic lines was obviously higher than that of *cbfs* triple mutants (Fig. 6e, Supplemental Fig. S3b). These results indicated that CeqCBF1 and CeqCBF3 dramatically enhance the cold tolerance of transgenic plants, though signature2 domain in CeqCBFs was different from that in AtCBFs.

### CBF binding motif analysis in promoter region of cold-induced genes in *C. equisetifolia*

CBFs, vital transcription factors in plant cold acclimation, promote the expression of a subset of cold responsive (*COR*) genes by specific binding to a CRT/DRE (G/ACCGAC) element in their promoters^[4,5]^. We identified the downstream target genes of CBFs from the transcriptome of both *A. thaliana* and *C. equisetifolia*, and subsequently analyzed the binding motif contained in *COR* genes' promoter region.

**Figure 7:**
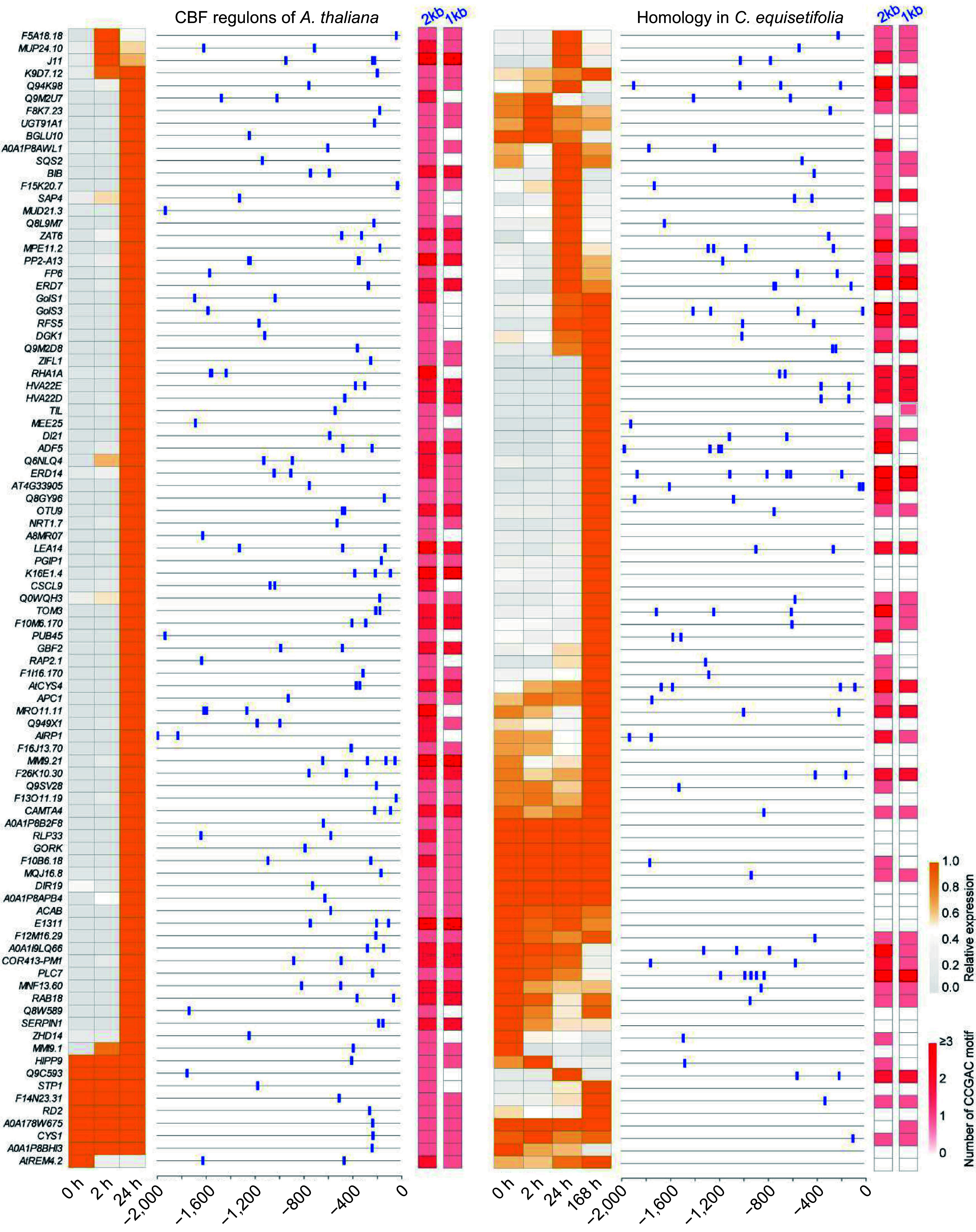
Heatmap and CRT/DRE element analysis of downstream cold response genes directly regulated by CBF in *A. thaliana* and *C. equisetifolia*.

For *A. thaliana,* we identified 91 downstream target genes of CBFs in transcriptome^[47]^, and most of them were highly induced by cold stress. Taken them as query, the same number homologous genes in *C. equisetifolia* were also identified. According to the transcriptome data, only half of the homologous genes in *C. equisetifolia* were up-regulated under low temperature condition, and a majority of the up-regulated genes exhibited a delayed response (Fig. 7). To investigate the CBF‐binding motif, the sequence of promoter located 1,000−2,000 bp upstream of ATG were analyzed by using FIMO^[49]^. In the 2,000 bp region, all genes contained CRT/DRE elements in *A. thaliana*, but only 58 of 91 contained CRT/DRE core motifs in *C. equisetifolia*. Moreover, the number of CRT/DRE binding motifs in the promoter of *COR* genes in *C. equisetifolia* was less than that in *A*. *thaliana* (Fig. 7), suggesting that the reduced number of CRT/DRE elements in the promoter region of *C. equisetifolia* may be associated with the delayed induction of *COR* genes in response to low temperature. Additionally, only half of the homologous genes in *C. equisetifolia* were up-regulated under cold treatment further supported the potential link between the decrease in CRT/DRE motifs and the reduced number of up-regulated genes in the downstream targets of CBFs in *C. equisetifolia*.

**Figure 8:**
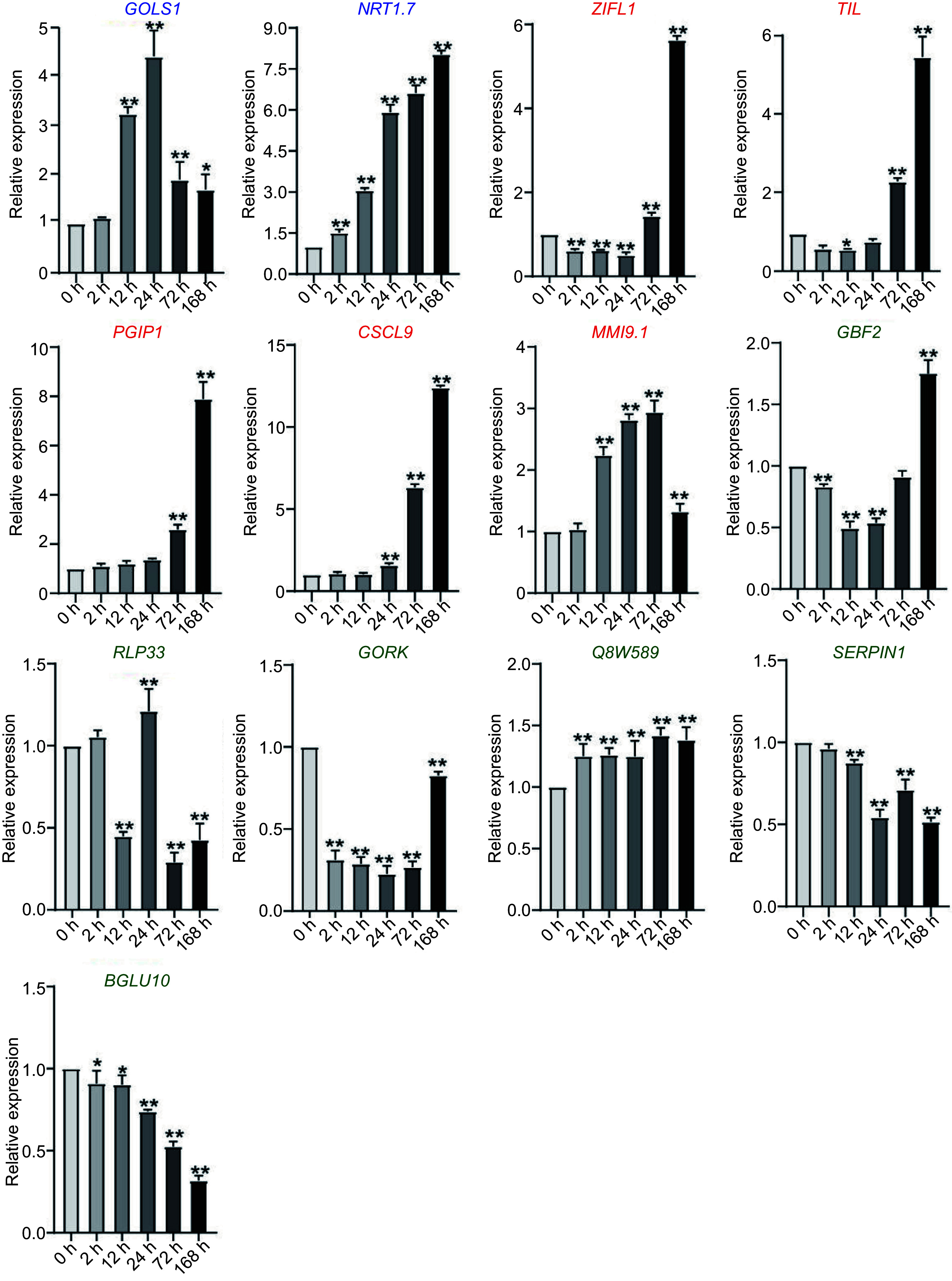
The expression of downstream genes without DRE elements but directly regulated by *CBF* in *C. equisetifolia*, containing two similar expression pattern genes (*GOLS1*, NRT1.7), five delayed genes (*ZIFL1*, *TIL*, *PGIP1*, *CSCL9*, *MMI9.1*) and six weakly responsive and non-responsive genes (*GBF2*, *RLP33*,*GORK*,*Q8W589*, *SERPIN1*, BGLU10), *GOLS1*, galactinol synthase 1; NRT1.7, NITRATE TRANSPORTER 1.7; ZIFL1, ZINC INDUCED FACILITATOR-LIKE 1; *TIL*, temperature-induced lipocalin; *PGIP1,* POLYGALACTURONASE INHIBITING PROTEIN 1; *GBF2*, G-box binding factor 2; *RLP33*, receptor like protein 33; *GORK*, GATED OUTWARDLY-RECTIFYING K^+^ CHANNEL; *SERPIN1*, Inhibitor of pro-apoptotic proteases, which is involved in the regulation of the programmed cell death induction; *BGLU10*, BETA GLUCOSIDASE 10. The values are the mean ± standard deviation of three biological replicates. Relative expression in untreated plants (0 h) was set to 1. *p < 0.05, **p < 0.01.

To prove this hypothesis, the expression of 13 genes without CRT/DRE element were detected in *C. equisetifolia* under cold stress. Of the genes, only two (*GOLS1* and *NRT 1.7*) genes were significantly increased at the 24 h time point under low temperature treatment, which was similar to that in *A. thaliana* (Fig. 8). The expression of five genes (*TIL, ZIFL1, PGIP1, CSCL9* and *MMI9.1*) was increased by cold, but obviously delayed in comparison with that in *A. thaliana* (Fig. 8). Additionally, the transcriptional levels of six genes, including *GRF2*, *RLP33*, *GORK*, *Q8W589*, *SERPIN1*, and *BGLU10*, did not show any obvious change under cold treatment (Fig. 8). These results implied that the absence of CRT/DRE elements resulted in either non-response or postponed response in the expression of cold induced genes, resulting in the weak cold tolerance in *C. equisetifolia*. We also determined the expression of the CBF downstream genes containing DRE elements in *C. equisetifolia*, including *ERD7*, *RD26*, *EXL2*, *HVA22D*, *LEA14*, and *RAV1*. These genes exhibited induced but delayed expression under cold stress (Supplemental Fig. S4), indicating that CRT/DRE elements were contributed to their expression mediated by CBF.

### The expression analysis of *CBF* upstream genes

**Figure 9:**
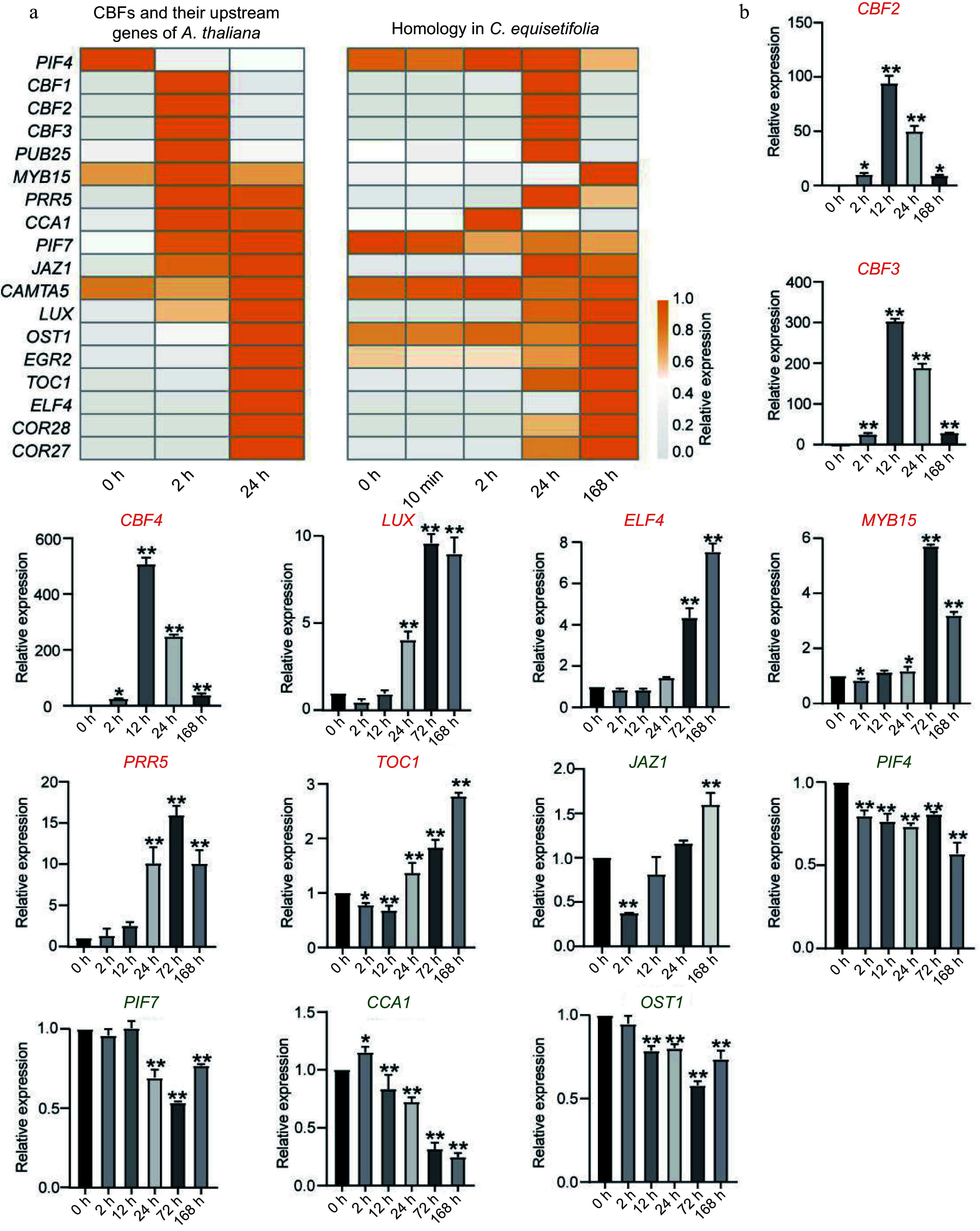
Comparison of upstream regulatory genes for *CBF* in *A. thaliana* and *C. equisetifolia*. (a) Heatmap of *CBF* upstream regulatory genes in *A. thaliana* and *C. equisetifolia*. (b) The expression of *CBF* upstream gene in *C. equisetifolia.* *CBF*, C-repeat/DRE binding factor; *LUX*, Homeodomain-like superfamily protein; *ELF4*, early flowering-like protein; *MYB15*, myb domain protein 15; *PRR5*, two-component response regulator-like protein; *TOC1*, CCT motif -containing response regulator protein; *JAZ1*, jasmonate-zim-domain protein 1; *PIF4/7*, phytochrome-interacting factor4/7; *CCA1*, circadian clock associated 1; *OST1*, Protein kinase superfamily protein. Relative expression in untreated plants (0 h) was set to 1. *p < 0.05, **p < 0.01.

It has been reported that cold stress rapidly up-regulated the transcriptional expression of the *CBFs* through a complex signal transduction network in *A. thaliana*, involving transcription factor ICE1, CAMTAs, MYB15, Circadian Clock‐related components CCA1/LHY, and the light signaling components PIF3/4/7^[6,62]^. Based on transcriptome analysis, we discovered a stark contrast in the expression patterns of *CBF* genes between *A. thaliana* and *C. equisetifolia* under cold treatment. In *A. thaliana*, the expressions of three *CBF* genes were significantly upregulated at 2 h after the start of cold treatment. However, in *C. equisetifolia*, the noticeable upregulation of their homologous genes was delayed and occurred after 24 h of cold treatment (Fig. 9a). Besides that, few upstream genes of *CBFs* were identified and their expression profiles were compared to that of *A. thaliana.* These upstream genes could be divided into three types according to their expression pattern: the first group including genes non-induced by cold stress (*PIF4*, *PIF7*, *CAMTA5*, *OST1*), the second group including delayed induced genes (*PUB25*, *MYB15*, *PRR5*, *JAZ1*, *LUX*, *EGR2*, *ELF4*, *COR28*, *COR27*) while the third group including genes with similar expression patterns to those in *A. thaliana* (*CCA1*, *TOC1*) (Fig. 9a). Further, the transcription levels of these genes and three *CeqCBFs* were detected under chilling treatment at different time points using qPCR. The results displayed that compared to the expression of genes in *A. thaliana,* the obvious increase in the expression of *CeqCBFs* (*CeqCBF1*, *CeqCBF2*, *CeqCBF3*, *CeqCBF4*, 2 h vs 12 h), *LUX* (24 h vs 72 h), *ELF4* (24 h vs 168 h), *MYB15* (2 h vs 72 h), *PRR5* (24 h vs 72 h), *TOC1* (24 h vs 168 h) was obviously delayed (Figs 5b, 9b). Chilling treatment did not induce the expression of *JAZ1*, *PIF4*, *PIF7*, *CCA1*, and *OST1*. These results demonstrated that one of the factors for the cold stress sensitivity of *C. equisetifolia* could be delayed response or non-response of the upstream regulatory genes.

### Analysis on the cold intolerant mechanism in tropical and subtropical plants

**Figure 10:**
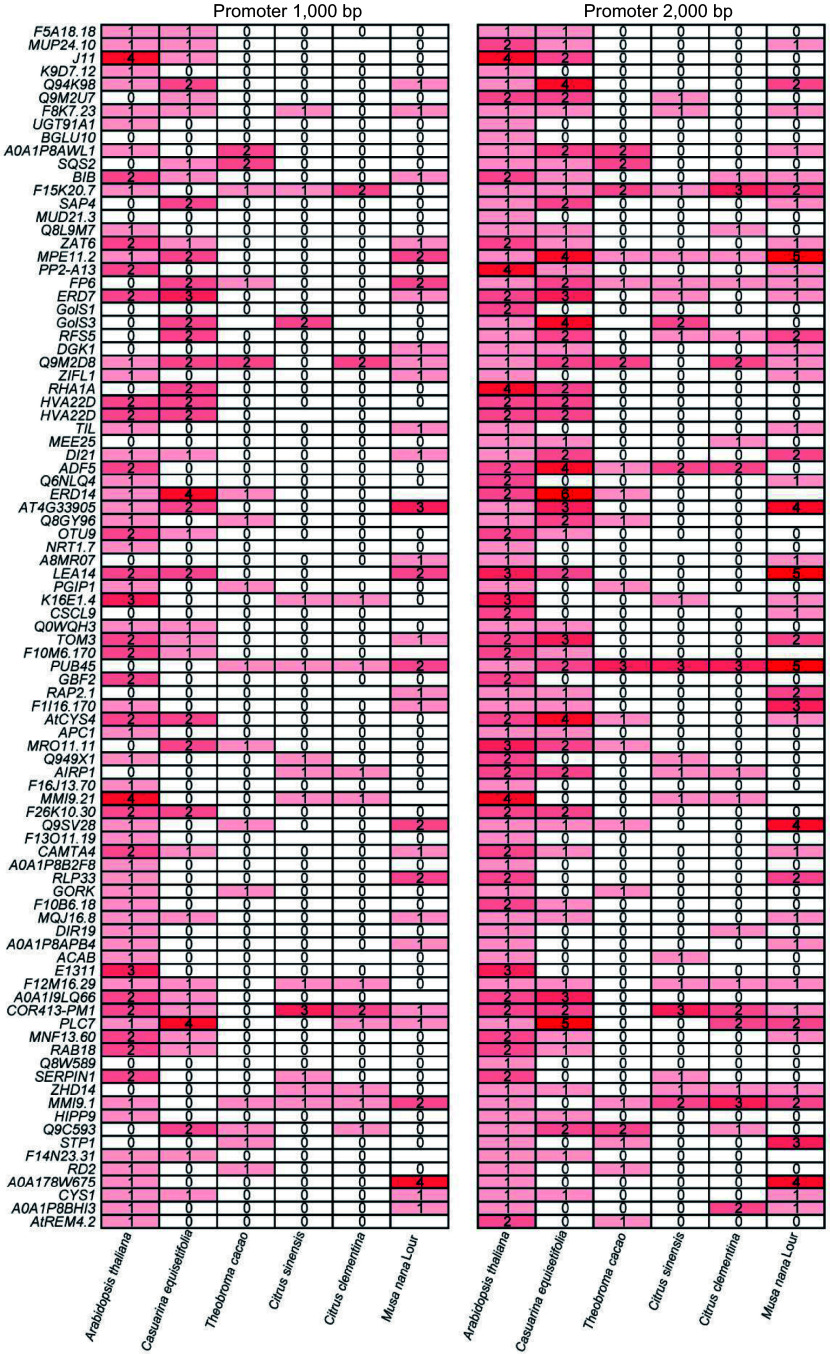
CRT/DRE element analysis of downstream cold response genes directly regulated by CBF in other tropical and subtropical plants.

*C. equisetifolia* is one of the typical tropical and subtropical plants, exhibiting sensitivity to cold stress, which is also the property of most of the tropical and subtropical plants. According to the results of our analysis, the absence of CBFs binding motif located in the promoters of their downstream genes maybe one of the main reasons for *C. equisetifolia*' cold intolerance (Fig. 7). To investigate whether the loss of CRT/DRE elements is common to tropical and subtropical plants and how it affects the cold response, the homologous proteins of CBFs in different tropical and subtropical plants, including *Theobroma cacao*, *Citrus sinensis*, *Citrus clementina*, and *Musa nana* Lour, *Citrus clementina*, *Citrus sinensis*, *Populus trichocarpa*, and *Hevea brasiliensis*, were identified by MEGA11 and phylogenetic tree was constructed. The result of CBF conserved domains analysis showed that typical domains of CBFs in these homologous proteins were highly conserved (Supplemental Fig. S5), indicating that CBFs may play similar roles in response to cold stress in different tropical and subtropical plants. Further, CRT/DRE motif was analyzed in CBF's downstream target genes by using FIMO^[49]^. The number of CRT/DRE motif was significantly reduced in the detected promoter range of downstream genes in different species (Fig. 10), demonstrating that absence of CRT/DRE may be a common feature for tropical and subtropical plants.

## Discussion

Cold acclimation is a vital strategy for improving plant cold resistance. Many tropical/subtropical plants, such as *Litchi chinensis*^[63]^, *Ananas comosus*^[64]^, *Citrus*^[65]^, *Musa spp*^[66]^, and *C. equisetifolia*^[67]^, can gradually distribute and survive in wider geographic and temperature ranges through cold acclimation. In this study, whether acclimated or not, *C. equisetifolia* maintained a low relative ion leakage rate when exposed to −2 °C, −4 °C, and −6 °C for 2 h, respectively (Supplemental Table S3), demonstrating that *C. equisetifolia* had a certain degree of tolerance to freezing stress for short treatment time. However, with longer exposure to low temperature, the ion leakage rate dropped to 17.16% after treatment at −6 °C for 6 h, compared to 53.89% of non-acclimated *C. equisetifolia* seedlings. On the contrary, it was prominent difference in the survival rate between the NA plants (21.15%) and CA plants (59.02%) (Supplemental Table S3). Further, when treating plants with the following condition (at 4 °C for 4 d and then −8 °C for 6 h), the rate of survival and the ion leakage of *C. equisetifolia* seedlings was separately 5.22% and 45.84% (Supplemental Table S3, Fig. 1c, d). In contrast, under the same treatment condition, the survival rate of *A. thaliana* increased from 0 to 100% after cold acclimation (Fig. 1c). This proposed that cold acclimation can improve the cold resistance of *C. equisetifolia* to some extent although this improvement was significantly lower than that of *A. thaliana*. It is speculated that *C. equisetifolia* may lack certain low temperature response or regulatory factors that are present in *A. thaliana*.

Comparing the low temperature transcriptomes of *C. equisetifolia* and *A. thaliana* showed that many low temperature responsive genes in *C. equisetifolia* exhibited no expression or weak or delayed response under low temperature stress, particularly in the CBF regulation pathway (Figs 4, 7 & 9). Further analysis revealed that numerous downstream target genes of CBF lacked the DRE binding element (Figs 7, 10), leading to influence the binding of CBFs to the downstream genes and then effect the expression of downstream genes, therefore decrease the cold adaptability of tropical and subtropical plants.

**Figure 11:**
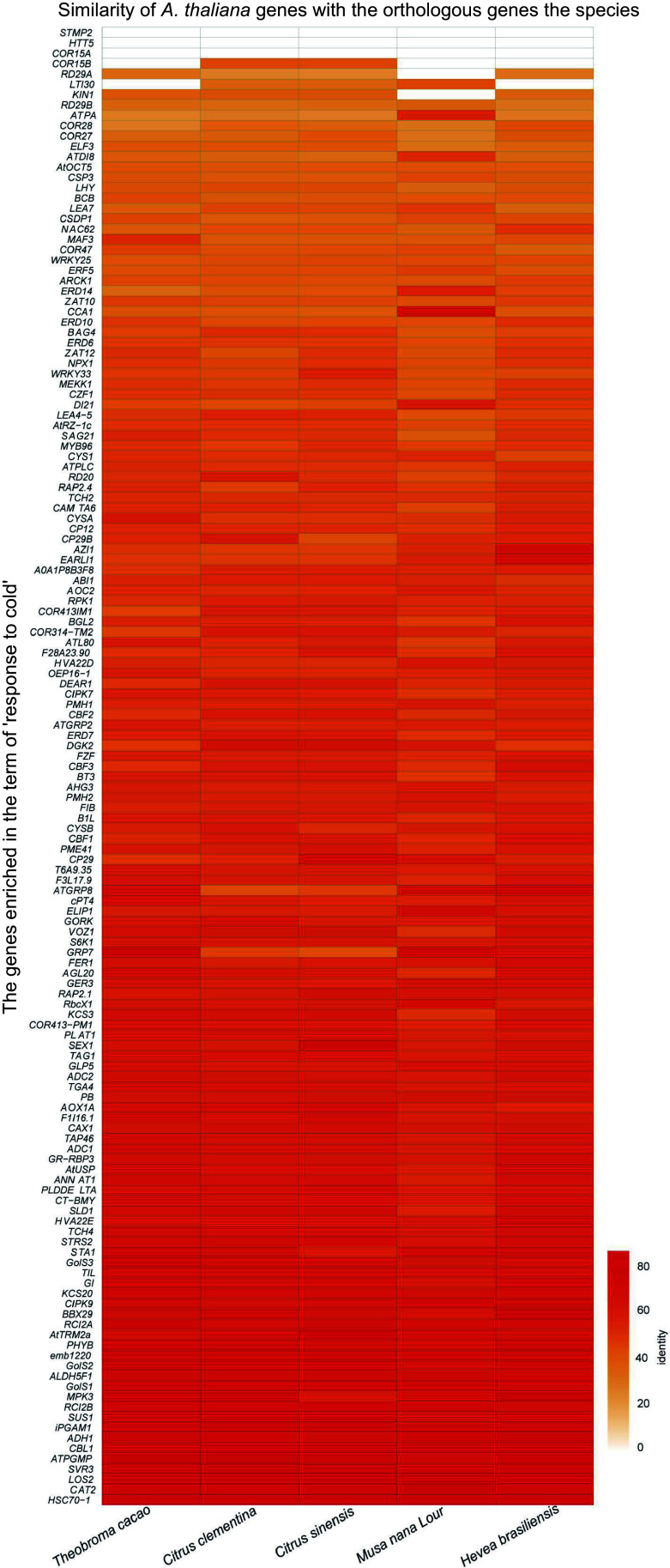
Homologous cold response genes in *A. thaliana* and other tropical/subtropical plants.

In *A. thaliana*, cold acclimation triggers altering the expression of a large number of cold regulated genes^[62]^. According to whether directly regulated by CBF or not, these *COR* genes are divided into CBF-dependent pathway and CBF-independent pathway. Many studies have reported that different transcription factors and proteins are involved in CBF-dependent signal pathways^[6]^. It has been shown that plenty of *COR* genes, including *KIN1/2*, *COR15A/B*, *LTI78*^[68,69]^, play major roles in regulating plant' cold response. Overexpressing of *COR15A*, encoding a chloroplast-targeted polypeptide in *A. thaliana*, results in a marked increase in the survival rate of separated protoplast frozen at −4.5 °C to −7 °C^[70,71]^. By comparing the homologous *COR* genes in *C. equisetifolia* and *A. thaliana*, we found that *C. equisetifolia* lacked homologous genes of *COR15A*, *COR15B,* *HTT5*, and *STMP2* (Fig. 4)*,* and the similar case was also discovered in other tropical/subtropical plants, such as *Theobroma cacao*, *Musa nana* Lour, *Citrus clementina*, *Citrus sinensis*, and *Hevea brasiliensis* (Fig. 11), speculating that the loss of key *COR* genes may be a crucial factor for their low temperature sensitivity and this may happen during the evolution process of tropical/subtropical plants in high temperature environments.

Cold-induced transcription factors CBF/DREB1 from AP2/ERF gene family respond to low temperature condition by directly modulating the expression of *COR* genes^[47,59]^. In *Arabidopsis*, the expression of *CBFs* shows rapid and early response to cold acclimation, and reaches the highest transcription level at 4 °C within 2 h^[61,72]^. Some studies have displayed that the expression of *CBFs* often exhibits a delayed response in *Hevea brasiliensis*, which is one of the typical tropical trees and sensitive to low temperature^[32,34,73]^. *C. equisetifolia* contained five *CBF* homologous genes, and cold acclimation highly induced the expression of *CeqCBFs*, which was accordance with the expression pattern of *CBFs* in *A. thaliana*, except that the peak expression of *CeqCBFs* occurred at 12 h after the start of cold acclimation (Fig. 5, Fig. 9b). In addition, CBF in *C. equisetifolia*, as well as in other tropical/subtropical plants, shared high conservation with *Arabidopsis* CBF in conserved structure domains, despite of certain differences in individual amino acid sites (Fig. 6a, Supplemental Fig. S5), suggesting that CBF maintained the conservation during the evolutionary process in plants at different latitudes. Nevertheless, our experiments had revealed that *C. equisetifolia* CBF could effectively restore the sensitive phenotype of *Arabidopsis cbf1/2/3* triple mutants to low temperature conditions (Fig. 6d, Supplemental Fig. S3a), indicating that CBFs were functionally conserved in *C. equisetifolia*. Considering that cold acclimation may take a relatively long time, the postpone response of *CeqCBFs* may not be the primary factor causing its sensitivity to low temperatures. Therefore, the main reason for the cold intolerance of *C. equisetifolia* could be the absence of the CBF binding element (DRE) of the *COR* genes in *C. equisetifolia* and multiple tropical/subtropical plants (Fig. 7, Fig. 11). We speculated that long-term exposure to high-temperature environments may result in the loss of these elements during the evolutionary process of tropical/subtropical plants, thereby, leading to the sensitivity to low temperatures. It is worth noting that, both in *C. equisetifolia* and *A. thaliana,* *COR* genes-mediated by the low temperature at different time points exhibited a typical time-dependent cascade amplification. In detail, most early cold responsive genes quickly returned to the expression levels before induction, while other cold responsive genes started to respond (Fig. 2c). In such a situation, the delayed expression of *CBF* may affect this cascade regulation mode, and may also be one of the reasons for *C. equisetifolia*'s cold intolerance.

**Figure 12:**
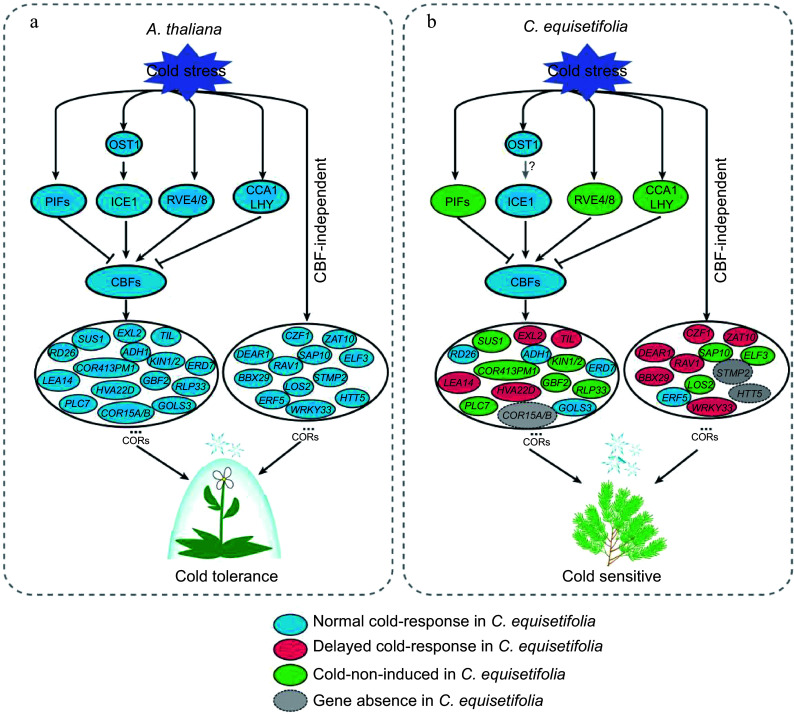
Comparative pattern plot of cold-response regulatory pathway in *A. thaliana* and *C. equisetifolia.*

Previous studies focused on the certification of low temperature responsive genes in order to understand the molecular regulation mechanisms and to enhance plant cold resistance through overexpression these genes. By comparing and analyzing differences in low-temperature response between the model plant *A. thaliana* and *C. equisetifolia*, we hypothesized that the loss of DRE elements in multiple *COR* genes in *C. equisetifolia* was associated with its response to low temperatures, and the absence of key *COR* genes was also a significant factor contributing to *C. equisetifolia*'s and other tropical/subtropical plants' sensitivity to low temperatures (Fig. 12). Therefore, introducing DRE elements into the related genes or expressing the absent genes in tropical/subtropical plants using modern biotechnological tools could be a new and important research ideas for improving the tropical/subtropical plants' cold resistance.

## SUPPLEMENTARY DATA

Supplementary data to this article can be found online.
