# Supplementary material for: Molecular mechanisms of low-temperature sensitivity in tropical/subtropical plants: a case study of Casuarina equisetifolia
Source: For Res (Fayettev). 2023 Aug 31;3:20. doi: 10.48130/FR-2023-0020 (PMC11524302; doi:10.48130/FR-2023-0020)
Supplement: Supplementary file 1 — Supplementary data to this article can be found online. [file FR-2023-0020-S1.zip › 10.48130_FR-2023-0020-Suppl-FigureS2.docx]

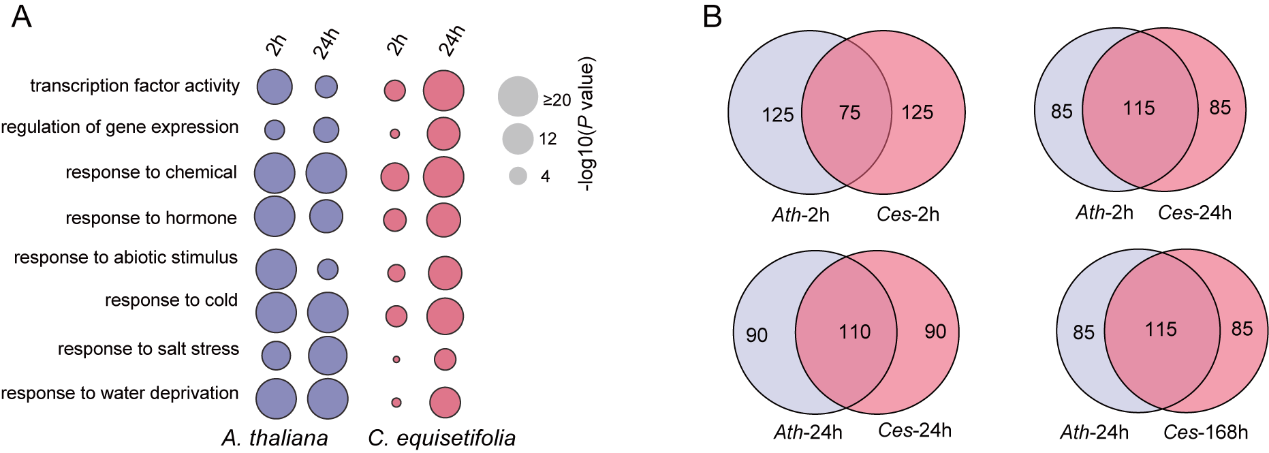


Fig. S2 GO entry enrichment analysis of cold-induced genes. **(A)** Presented in 2 h and 24 h mainly deferred entries. **(B)** Venn diagrams showing the overlap of the 200 most enriched entries with *A. thaliana* and *C. equisetifolia* after cold treatment for three time points.
