# Supplementary material for: Molecular mechanisms of low-temperature sensitivity in tropical/subtropical plants: a case study of Casuarina equisetifolia
Source: For Res (Fayettev). 2023 Aug 31;3:20. doi: 10.48130/FR-2023-0020 (PMC11524302; doi:10.48130/FR-2023-0020)
Supplement: Supplementary file 1 — Supplementary data to this article can be found online. [file FR-2023-0020-S1.zip › 10.48130_FR-2023-0020-Suppl-FigureS3.docx]

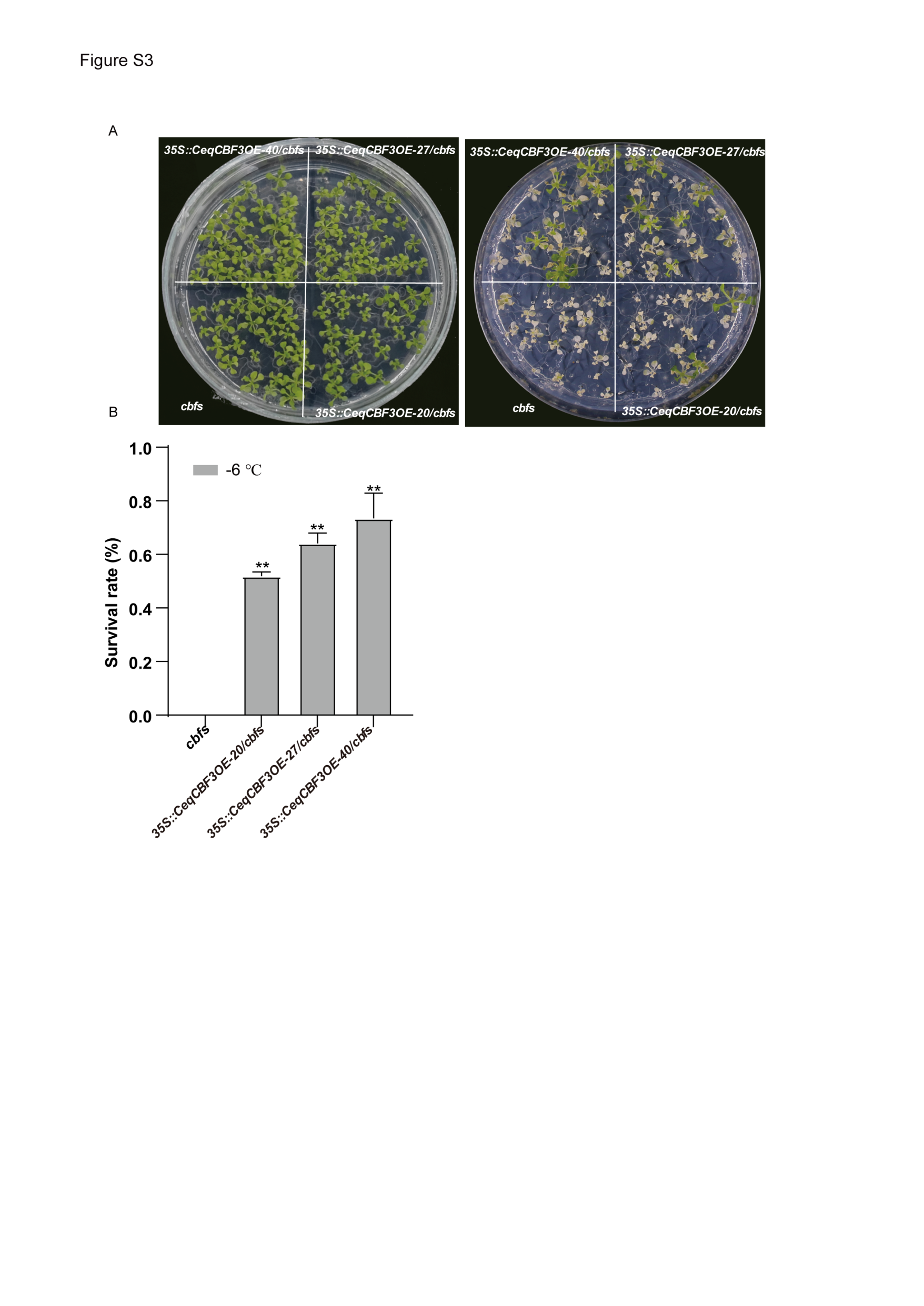


Fig. S3 CBF in *C. equisetifolia* was involved in cold stress regulation. Freezing phenotypes **(A)**, survival rates **(B)** of *A. thaliana* *cbf* triple mutants and transgenic lines with overexpressing of *CeqCBF3*. Two-week-old plants grown on MS plates at 22 ℃ were treated at -6 ℃ for 6 h after cold acclimation at 4 ℃ for 3 days. Asterisks represent significant differences compared to the *cbfs* mutant plants. ***P* < 0.01.
