# Supplementary material for: Molecular mechanisms of low-temperature sensitivity in tropical/subtropical plants: a case study of Casuarina equisetifolia
Source: For Res (Fayettev). 2023 Aug 31;3:20. doi: 10.48130/FR-2023-0020 (PMC11524302; doi:10.48130/FR-2023-0020)
Supplement: Supplementary file 1 — Supplementary data to this article can be found online. [file FR-2023-0020-S1.zip › 10.48130_FR-2023-0020-Suppl-FigureS4.docx]

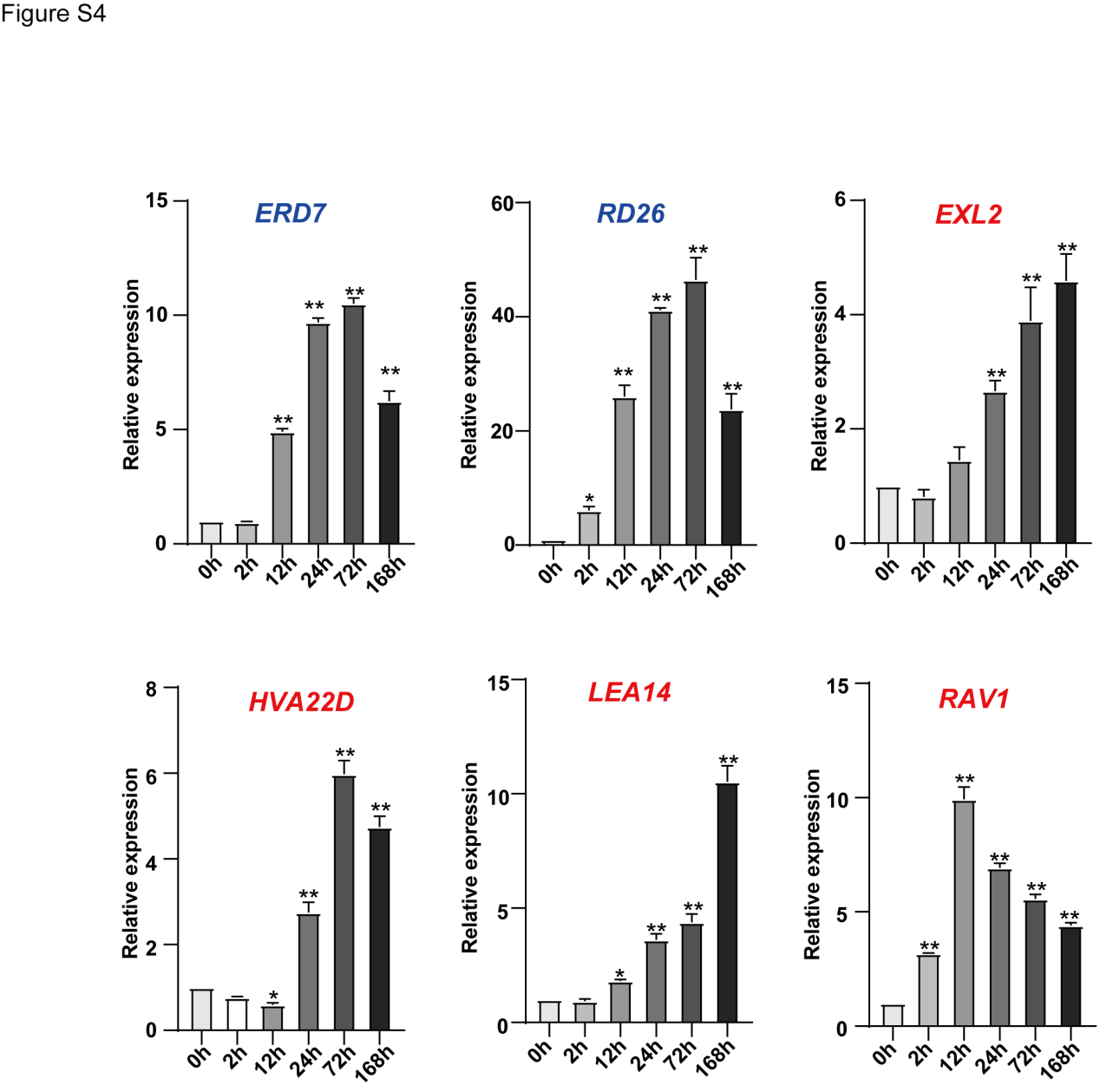


Fig. S4 The expression of downstream genes directly regulated by *CBF* in *C. equisetifolia*. *ERD7*, Early Responsive to Dehydration 7; *RD26*, RESPONSIVE TO DESICCATION 26; *EXL2*, EXORDIUM LIKE 2; *HVA22D*, *HVA22* homologue D; *LEA14*, LATE EMBRYOGENESIS ABUNDANT 14; *RAV1*, ETHYLENE RESPONSE DNA BINDING FACTOR 4. The values are the mean ± standard deviation of three biological replicates. Relative expression in untreated plants (0 h) was set to 1. **P* < 0.05, ***P* < 0.01.
