# Supplementary material for: Molecular mechanisms of low-temperature sensitivity in tropical/subtropical plants: a case study of Casuarina equisetifolia
Source: For Res (Fayettev). 2023 Aug 31;3:20. doi: 10.48130/FR-2023-0020 (PMC11524302; doi:10.48130/FR-2023-0020)
Supplement: Supplementary file 1 — Supplementary data to this article can be found online. [file FR-2023-0020-S1.zip › 10.48130_FR-2023-0020-Suppl-TableS1.docx]

**Table S1. Primer for Real-Time quantitative PCR**

| **Gene name** | **Forward primer sequence（5’-3’）** | **Reverse primer sequence（5’-3’）** |
| --- | --- | --- |
| Casgl152S09377(*ERD14*) | GGAAGAGGAGACCAAGGACC | ACCGTGTTTCTTCTCGTCCT |
| Casgl30S01239(*DEAR1*) | TGAGGAAGTGGGGAAAGTGG | CCTTGGAGAAGCAAATCGGG |
| Casgl31S15525(*ELF3*) | GTACTGTCCACACCGTCTGA | TCTTGGCCCTCCTTTCTCTG |
| Casgl31S15535(*MPK3*) | CTGATGACTCCAGCCTAGGG | CTTGGGCAAACAGGTTCCTC |
| Casgl33S01650(*DGK2*) | AAGCTACAAGATGCCCCAGT | CCATGTTGACCTCCACTCCT |
| Casgl461S18355(*KIN2*) | CTTTCAGGCTGGGGAGGC | GACGGACTCAGCAGCTTCTT |
| Casgl53S19451(*RD29A*) | CGAGCTATAATGGCCAGGGA | GCAGCGTTACTACTTGAGCC |
| Casgl602S25733(*ZAT10*) | AGGAGATGGATAGGCAGTGC | TTGTCTCTCCCGTTCAGCTT |
| Casgl64S20644(*ADH1*) | ACCAAACAAGGACGATGCAT | CTTTGTGCCTTTCCCTTGCA |
| Casgl71S21325(*WRKY33*) | CGCACCCAACAAGCTTTACT | CACACCTGACCCTGTTCTCT |
| Casgl72S21481(*GolS1*) | CCCTCATCCTCATCCTCGAT | ATCGGGAGCCATTTGAAGTG |
| Casgl8S02712(*DREB26*) | GGGAGAGGATTTGGTTGGGA | CCTGAATCTCGGACCTGGAG |
| Casgl82S22459(*LOS2*) | CCTCCGATCCAGTCCTCTTC | CACGTATTTCAAGGGCCTCG |
| Casgl9S23018(*BBX29*) | TGTTGGGACTGTGACGCTAT | TTATTTCTGACGCAGCGCTC |
| Casgl94S23270(*CBL1*) | GGGTACGAGGATCCTGTTGT | ATAACCCCATTGCGCTTCAC |
| Casgl96S03010(*CIPK7*) | CAAGAAGATTCACCGCCGAG | ACATGTCGTTCTTGGTGCAC |
| Casgl1065S06900(*RD26*) | TCAACTCGGGACCTGAACTC | TCCTGAGTTTTCCATCCGCT |
| Casgl1249S07727(*ERD7*) | AGATTAGCCCGGGAGTGATG | ACATGACATTCCTCCCAGCA |
| Casgl20S11083(*HVA22D*) | GGATGACGAACAGTGGCTTG | AAGAAAGCTGCCCCTTGGAA |
| Casgl22S11549(*COR413-PM1*) | CGCTTCCTTCGCTGCTATTT | GGGAAGTGTCGAGGGAAGAA |
| Casgl43S05246(*EXL2*) | CTTCCATCCGCTTCATCGTG | AACAGCAACATCCTTGGCAG |
| Casgl437S05274(*PLC7*) | CTCGCTCGCTGATTTCTTCC | CTCTGCAACGCCCTAATGAC |
| Casgl64S02381(*RAV1*) | ACTTCCCTCTACAAAGCGCT | TGTAAAGTTGCTTGTCCGGC |
| Casgl72S21414(*GOLS3*) | ATCAGCTACCAAACCCGTGA | CGAACTGGGTCTGGTTTTCG |
| Casgl731S05936(*TIL*) | ACGAGGCAAAGCTCAAAGTG | TTGCCTGCACAGTATCCAGA |
| Casgl78S21965(*GBF2*) | ACCTGCTGCTGTAACTCCTT | AGTCTCCAAACTCTGCGACA |
| Casgl78S21899(*LEA14*) | TGTCCGTGAAGAACCCCTAC | CAATTGTGAGGCCCAGTTCC |
| Casgl8S22188(*SUS1*) | TCGAAGCAATCCCTGAGGTT | AAATTCCCGTTGGCACTTCC |
| Casgl53S05669(*CBF1*) | GACGTTCCCAACTCCTGAGA | TCCGGCGATTCTCTCTTCTC |
| Casgl344S25205(*CBF2*) | AAGTTCAAGGAGACGAGGCA | CCGATTCTGCGAAGTTGAGG |
| Casgl344S16225(*CBF4*) | TCAGTATTCGTCCGAGTCCG | AGTTCCTCCTCCTCACTCCT |
| Casgl72S21531(*EGR2*) | CCAGCAGAGACCAATTCGTG | ATTTGCGGAAGGGAAGGAGA |
| Casgl782S06033(*PUB25/26*) | CAGTGGGATGCTCTGCTTTC | CCGGAGAAGCTTCAGGAGAA |
| Casgl158S09591(*MYB15*) | AAAGTTCAAGCGACGTGTCC | GGTACTTCCCCTGCTCTTGT |
| Casgl8S22205(*JAZ1*) | GTGACTAGCTCTACTGCCGT | GGTTGCTTGTTTGGTACGGT |
| Casgl177S10384(*TOC1*) | GGAATTAGTGACCGGCGAAC | CTGCCTGCCCATTTTCATGT |
| Casgl437S01986(*ELF4*) | TGACCTCACCCGATTCTTCC | GCCACGTTCTTCACCATGTT |
| Casgl102S00066(*LUX*) | GCGGTGCCCAAGACAATTAT | GATACGGCATCTGAATCGGC |
| Casgl94S23335(*PRR5*) | TGGTGAGGTGGGAGAAGTTC | TCTCCCATGCCTTTAAGCCA |
| Casgl244S12439(*CAMTA5*) | TACCACAAGCTGCTGGATCA | TTGGAGGCTTTTGGACGTTG |
| Casgl196S10951(*OST1*) | TCTCCAGAGTGTCGCCATTT | TTTGCAAGGGTTGATCAGGC |
| Casgl447S18096(*PIF4*) | GTGATGCAGAGCCAAAACCA | GGTGCTGTCTAAGGAAGGGT |
| Casgl1071S06937(*PIF7*) | GAGTGCGAGAAACATGTCCC | CGAATGGAGGCGAAACGAAT |
| Casgl31S15258(*CCA1/LHY*) | TGCATCGCTGTTAGACCTCA | TGGGACATTTCTCTTCGCCT |
| CCG026826(*ACAB*) | TTCCACAACTGCTAGGGTGT | CTTCGCACCAAGGCCTTATC |
| CCG026854(*Q8W589*) | CTCCTCCTCCTCCTCCTCAT | CCGACTAACCAGGTCCATCA |
| CCG014049(*MUD21.3*) | TGCCTGAAAGCTGTTCCAAC | AGACGCTTTGCACTCAGTTG |
| CCG015264(*NRT1.7*) | TTTGCCGAAAGAAGAGGTGC | CCCCGTTTGTTGTTCGATGA |
| CCG017734(*GORK*) | GAGGAGGGGTTGGTGATGAA | ATGAGCATCCAGTCTGAGGG |
| CCG020525(*RLP33*) | TTCTCCCCTCACTCAACACC | AATCCACCCCATCCCAAGAG |
| CCG021678(*MMI9.1*) | CTCGCTCAACCACTTCCATG | CAAAGCAGCACTAACCCAGG |
| CCG002360(*SERPIN1*) | TGTTCTCGCCGCTTTCAATC | ACTTATCGAGCCAAACCCCA |
| CCG008885(*ZIFL1*) | CATCCCCTGCGTCTAATGGA | ACTTCCCCGACATCATTGGT |
| CCG010120(*BGLU10*) | GCGCGGACTTATTGGAATCA | GAAGGCCGGAAGTCTTGTTC |
| CCG011933(*CSCL9*) | TGAGAAGGACAGTGTTGGGG | GAAGAACCTGTGCAGCAACA |
| CCG012286(*PGIP1*) | CGCAAGCTATCCAACCTCAC | AGAGGTTGTTGAAGGCGAGA |
| *CeqUBC*-ACTIN | CCATGGTGTACTCTCTCTGCC | TGTTTTGTGATAAGGATTACCCAGA |
| pCAMBIA1300-GFP | AAGGAAAGGCCATCGTTGAAG | TCTTGTAGTTGCCGTCGTCC |
