# Supplementary material for: Molecular mechanisms of low-temperature sensitivity in tropical/subtropical plants: a case study of Casuarina equisetifolia
Source: For Res (Fayettev). 2023 Aug 31;3:20. doi: 10.48130/FR-2023-0020 (PMC11524302; doi:10.48130/FR-2023-0020)
Supplement: Supplementary file 1 — Supplementary data to this article can be found online. [file FR-2023-0020-S1.zip › 10.48130_FR-2023-0020-Suppl-TableS2.docx]

**Table S2. Transgenic primer sequences**

| **Gene name** | **Forward primer sequence（5’-3’）** | **Reverse primer sequence（5’-3’）** |
| --- | --- | --- |
| Casgl53S05669 (*CeqCBF1*) | TCCCCCCGGGATGGATGTCTTCTCTCAGTA | GCTCTAGATCAAATGGAAAAACTCCATAGTG |
| Casgl344S25208 (*CeqCBF3*) | TCCCCCCGGGATGGATGGCTTCTCTCAGTA | GCTCTAGATCAAATTGAGTAACTCCATAG |
| ATG42540(*COR15A*) | TCCCCCCGGGATGGCGATGTCTTTCTCAGG | GCTCTAGACTTTGTGGCATCCTTAGCCT |
