# Supplementary material for: Molecular mechanisms of low-temperature sensitivity in tropical/subtropical plants: a case study of Casuarina equisetifolia
Source: For Res (Fayettev). 2023 Aug 31;3:20. doi: 10.48130/FR-2023-0020 (PMC11524302; doi:10.48130/FR-2023-0020)
Supplement: Supplementary file 1 — Supplementary data to this article can be found online. [file FR-2023-0020-S1.zip › 10.48130_FR-2023-0020-Suppl-TableS3.docx]

**Table S3. Ion leakage rate and survival rate of *C. equisetifolia* under different temperature and time treatment**

| **Stress time（h）** | Stress temperature（℃） | **NA** | | **CA** | |
| --- | --- | --- | --- | --- | --- |
|  |  | Ion leakage (%) | Survival rate (%) | Ion leakage (%) | Survival rate (%) |
| 0 | 23 | 4.03 | \ | 6.40 | \ |
| 2 | -2 | 4.01 | \ | 7.14 | \ |
|  | -4 | 3.83 | \ | 5.55 | \ |
|  | -6 | 4.95 | \ | 5.76 | \ |
| 6 | -6 | 53.89 | 21.15 | 17.16 | 59.02 |
|  | -8 | 62.94 | 0 | 45.84 | 5.22 |

NA: non-acclimated; CA: 4℃ cold-acclimated 4 d
