# Supplementary figures and images for: Molecular mechanisms of low-temperature sensitivity in tropical/subtropical plants: a case study of Casuarina equisetifolia
Source: For Res (Fayettev). 2023 Aug 31;3:20. doi: 10.48130/FR-2023-0020 (PMC11524302; doi:10.48130/FR-2023-0020)

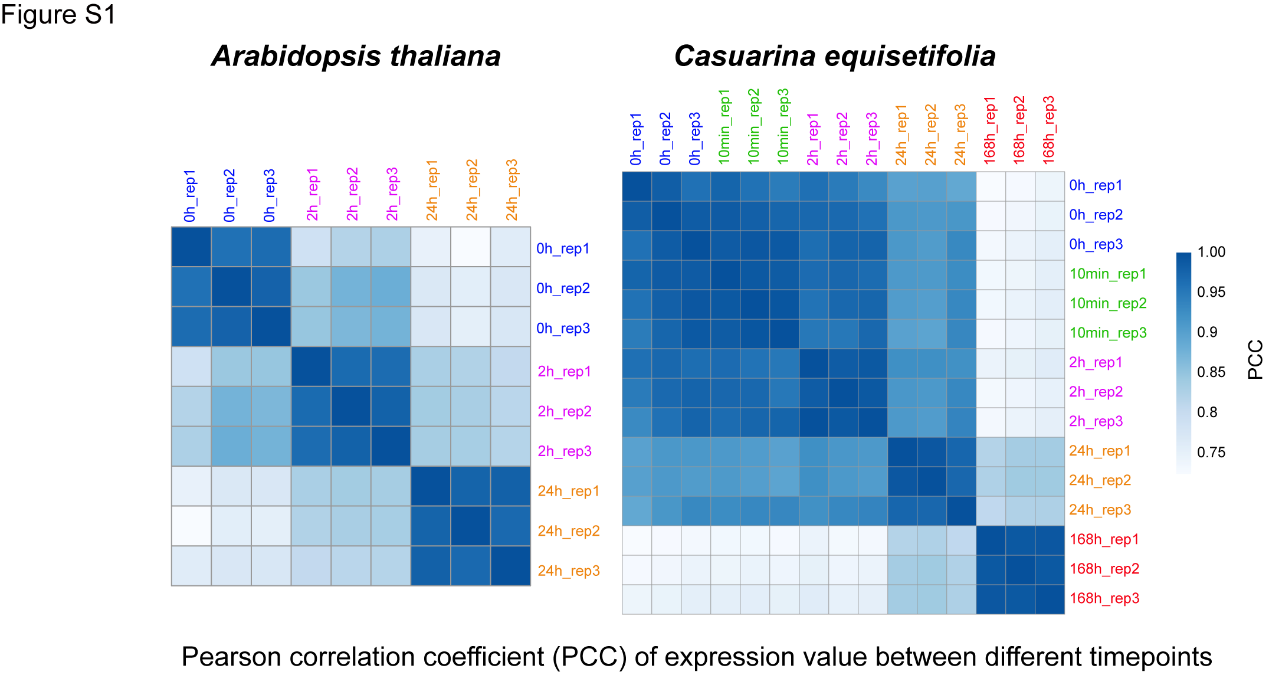


Fig. S1 Pearson correlation coefficient (PCC) of expression value at different time points.

Supplement: Supplementary file 1 — Supplementary data to this article can be found online. [file FR-2023-0020-S1.zip › 10.48130_FR-2023-0020-Suppl-FigureS1.docx]
